# Use of esophageal balloon pressure-volume curve analysis to determine esophageal wall elastance and calibrate raw esophageal pressure: a bench experiment and clinical study

**DOI:** 10.1186/s12871-018-0488-6

**Published:** 2018-02-14

**Authors:** Xiu-Mei Sun, Guang-Qiang Chen, Hua-Wei Huang, Xuan He, Yan-Lin Yang, Zhong-Hua Shi, Ming Xu, Jian-Xin Zhou

**Affiliations:** Department of Critical Care Medicine, Beijing Tiantan Hospital, Capital Medical University, Address: No 6, Tiantan Xili, Dongcheng district, Beijing, 100050 China

**Keywords:** Esophageal pressure, Esophageal balloon catheter, Esophageal elastance, Calibration, Bench experiment

## Abstract

**Background:**

Accurate measurement of esophageal pressure (Pes) depends on proper filling of the balloon. Esophageal wall elastance (Ees) may also influence the measurement. We examined the estimation of balloon-surrounding elastance in a bench model and investigated a simplified calibrating procedure of Pes in a balloon with relatively small volume.

**Methods:**

The Cooper balloon catheter (geometric volume of 2.8 ml) was used in the present study. The balloon was progressively inflated in different gas-tight glass chambers with different inner volumes. Chamber elastance was measured by the fitting of chamber pressure and balloon volume. Balloon pressure-volume (P-V) curves were obtained, and the slope of the intermediate linear section was defined as the estimated chamber elastance. Balloon volume tests were also performed in 40 patients under controlled ventilation. The slope of the intermediate linear section on the end-expiratory esophageal P-V curve was calculated as the Ees. The balloon volume with the largest Pes tidal swing was defined as the best volume. Pressure generated by the esophageal wall during balloon inflation (Pew) was estimated as the product of Ees and best volume. Because the clinical intermediate linear section enclosed filling volume of 0.6 to 1.4 ml in each of the patient, we simplified the estimation of Ees by only using parameters at these two filling volumes.

**Results:**

In the bench experiment, bias (lower and upper limits of agreement) was 0.5 (0.2 to 0.8) cmH_2_O/ml between the estimated and measured chamber elastance. The intermediate linear section on the clinical and bench P-V curves resembled each other. Median (interquartile range) Ees was 3.3 (2.5–4.1) cmH_2_O/ml. Clinical best volume was 1.0 (0.8–1.2) ml and ranged from 0.6 to 1.4 ml. Estimated Pew at the best volume was 2.8 (2.5–3.5) cmH_2_O with a maximum value of 5.2 cmH_2_O. Compared with the conventional method, bias (lower and upper limits of agreement) of Ees estimated by the simple method was − 0.1 (− 0.7 to 0.6) cmH_2_O/ml.

**Conclusions:**

The slope of the intermediate linear section on the balloon P-V curve correlated with the balloon-surrounding elastance. The estimation of Ees and calibration of Pes were feasible for a small-volume-balloon.

**Trial registration:**

Identifier NCT02976844. Retrospectively registered on 29 November 2016.

**Electronic supplementary material:**

The online version of this article (10.1186/s12871-018-0488-6) contains supplementary material, which is available to authorized users.

## Background

Esophageal pressure (Pes), which is commonly measured by a catheter with an air-filled balloon, has been used to estimate pleural pressure [1, 2]. Recently, this technique has regained attention to guide lung-protective ventilation in patients with acute respiratory distress syndrome [3–7]. An accurate measurement of Pes depends on the proper filling of the balloon [8–13]. Under in vitro conditions at atmospheric pressure, during progressive inflation of the balloon, the balloon pressure-volume (P-V) curve exhibits a nearly plain intermediate section, indicating a volume range with negligible balloon recoil pressure [8–11]. Nevertheless, when the balloon is placed in the esophagus, inflation of the balloon yields an inclined linear intermediate section on the P-V curve [8, 12, 13]. This phenomenon has been thought to be due to the response of the esophageal wall recoil to the balloon inflation, and the slope of the intermediate linear section is considered to be equal to the esophageal wall elastance (Ees) [8, 12, 13]. Although release-derived and elastance-derived strategies have been proposed to compute relative transpulmonary pressure [14], eliminating the influence of balloon surrounding structures on the absolute measurement of Pes may also be required [3, 4, 7]. Based on the estimation of Ees and pressure generated by the esophageal wall during balloon inflation (Pew), Mojoli and coworkers introduced an in vivo calibration procedure to make the Pes measurement more reliable [13]. However, to the best of our knowledge, no study has been performed to testify the certainty of this calibration method. Moreover, only one type of balloon with a relatively large geometric volume has been investigated [13].

In the present study, we established a bench model to simulate different levels of balloon-surrounding elastance and performed balloon volume test using a small-volume-balloon catheter. The balloon-surrounding elastance was estimated by the slope of the intermediate linear section on the balloon P-V curve. We primarily aimed to examine the agreement between the estimated and measured elastance. The balloon volume test was also performed in passive patients under controlled ventilation. Balloon P-V curves obtained in vitro and (in vivo conditions were compared. Based on previously introduced methods [12, 13], we developed a simplified procedure for the Ees estimation and Pes calibration. The secondary aims included the assessment of the agreement between the standard and simple methods, and the comparison of calibrated Pes values among different filling volumes.

## Methods

### Detailed methods are presented in Additional file 1.

A commercially available esophageal balloon catheter (Cooper: LOT 177405, Cooper Surgical, USA) was used in the present study [9–11]. During the study, pressure waveforms were measured and recorded by a dedicated data acquisition system [11].

### Bench experiment

**Fig. 1:**
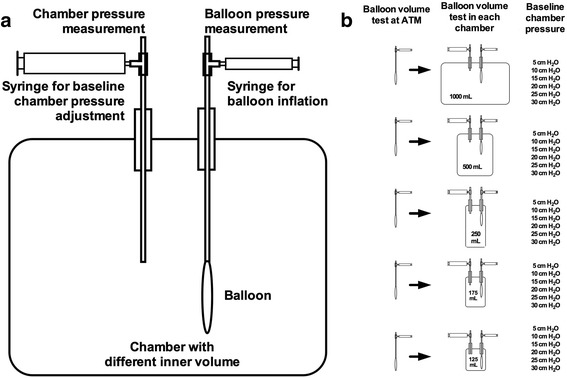
The bench model and experimental protocol. Panel (**a**) Each glass chamber had two openings: one for introducing the balloon into the chamber and the other one for adjusting and measuring the chamber pressure. Panel (**b**) Balloon volume tests were performed in five chambers with respective inner volume of 1000, 500, 250, 175 and 125 ml. In each chamber, baseline pressure was adjusted to 5, 10, 15, 20, 25 and 30 cmH_2_O. Before each balloon volume test, connections in the chamber system were sealed by silicone sealant, and systematic leaks were checked

Five glass chambers with an inner volume of 1000, 500, 250, 175 and 125 ml were used to obtain five levels of chamber elastance approximately from 1 to 8 cmH_2_O/ml (Fig. 1a) [9, 15]. In each chamber, six levels of baseline chamber pressure (5, 10, 15, 20, 25 and 30 cmH_2_O) were also established by injecting different amounts of air into the chamber. Thus, 30 balloon-surrounding conditions with five levels of chamber elastance and six levels of baseline chamber pressure were simulated (Fig. 1b).

Five Cooper catheters were selected and randomly introduced into the five different chambers. Before each balloon volume test, the residual volume of the balloon was standardized as the method described in previous studies [16–18]. Briefly, the chamber pressure was adjusted to 5 cmH_2_O by air injection and the balloon lumen was opened to the atmosphere for 3 min. We arbitrarily defined the balloon volume under this condition as the zero filling volume. Then the balloon was intermittently inflated in 0.2-ml increments up to 2.4 ml. A balloon volume test was first performed at atmospheric pressure and then in each chamber with certain elastance at different levels of baseline chamber pressure (Fig. 1b).

Balloon pressure and chamber pressure were simultaneously measured. Balloon transmural pressure was defined as the difference between the balloon pressure and the chamber pressure (balloon pressure - chamber pressure) [9, 10]. The balloon volume with transmural pressure within ±1.0 cmH_2_O was defined as the minimal and maximal balloon volumes (V_MIN_ and V_MAX_), which represented the optimal filling volume with the least influence of balloon recoil pressure [9, 10]. Balloon working volume (V_WORK_) was calculated as the difference between V_MIN_ and V_MAX_. The balloon volume with the closest to zero transmural pressure was defined as the best filling volume (V_BEST_).

**Fig. 2:**
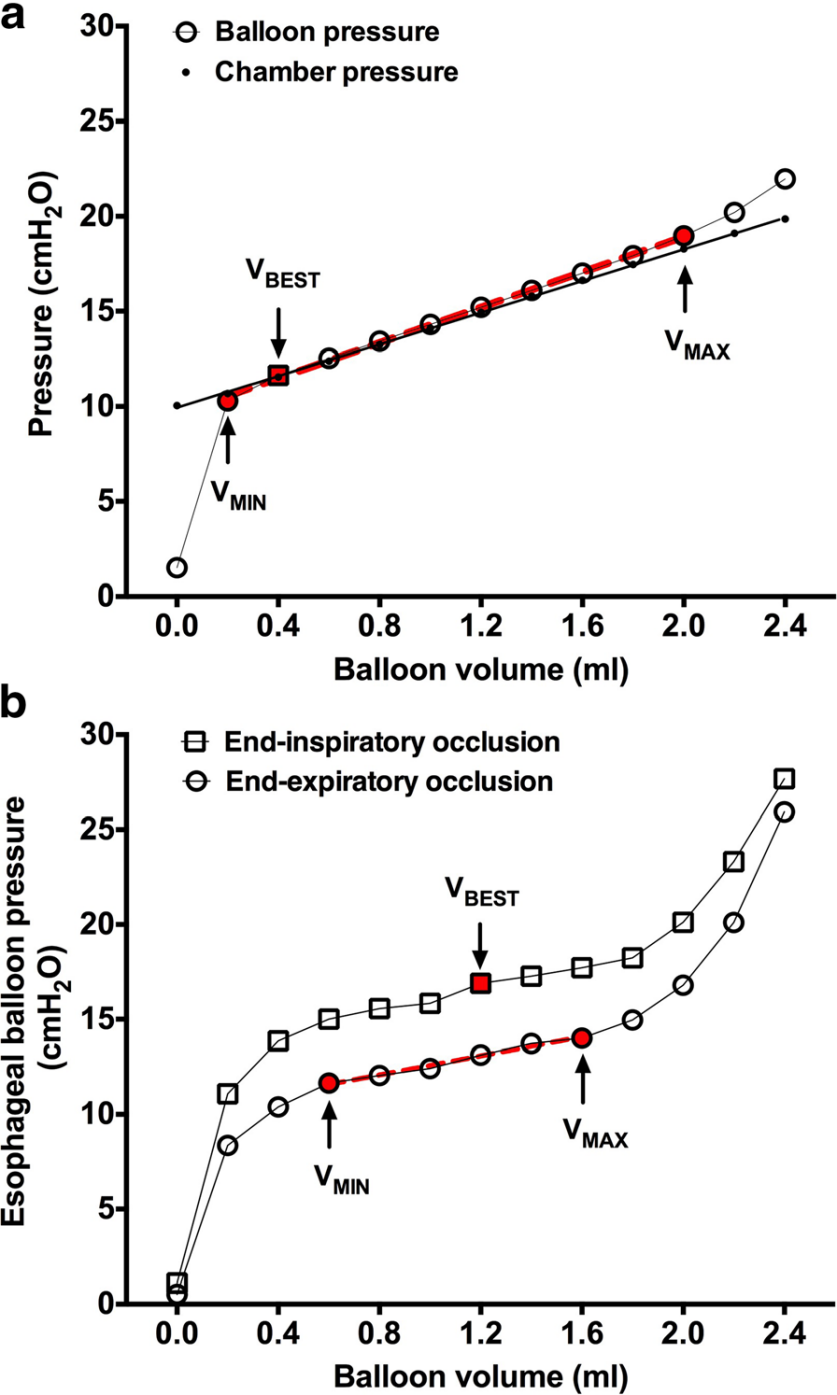
Examples of balloon pressure-volume curves. Panel (**a**) Balloon pressure (circles) and chamber pressure (dots) were plotted against balloon volume in the bench experiment. The balloon volume with transmural pressure (the difference between the balloon pressure and the chamber pressure) within ±1.0 cmH_2_O was defined as the minimal and maximal balloon volumes (V_MIN_ and V_MAX_). The balloon volume with the closest to zero transmural pressure was defined as the best filling volume (V_BEST_). Least square fitting line between the chamber pressure and balloon volume (black line) and between the balloon pressure and balloon volume within V_MIN_ to V_MAX_ (red dash line) are also shown. Panel (**b**) Esophageal balloon pressure at end-expiratory (circles) and end-inspiratory occlusion (squares) were plotted against balloon volume in the clinical study. The lower and upper limits of an intermediate linear section on expiratory balloon pressure-volume curve were visually inspected, and were defined as the clinical V_MIN_ and V_MAX_. The clinical V_BEST_ was defined as the balloon volume with the largest difference between end-expiratory and end-inspiratory esophageal balloon pressure. Least square fitting line between the end-expiratory balloon pressure and balloon volume within clinical V_MIN_ to V_MAX_ (red dash line) is also shown

Balloon pressure and chamber pressure were plotted against the balloon volume (Fig. 2a). The chamber pressure increased linearly as a function of balloon volume, and the slope obtained by least square fitting was defined as the measured chamber elastance. The balloon P-V curve exhibited a sigmoid shape with an intermediate linear section corresponding to the V_MIN_ to V_MAX_ range. We used the slope of this linear section to estimate chamber elastance. Baseline chamber pressure was estimated as the difference between the measured balloon pressure at V_BEST_ and the product of estimated chamber elastance and V_BEST_.

### Clinical study

The clinical study was conducted in the intensive care unit, Beijing Tiantan Hospital, Capital Medical University, Beijing, China. The study protocol was reviewed and approved by the local Institutional Review Board (KY-2016-11-22) and the study was registered at ClinicalTrials.gov (NCT02976844). Written informed consent was obtained from the patient or appropriate substitute decision makers.

We enrolled postoperative patients with delayed emergence from general anesthesia admitted to the unit for mechanical ventilation. For patients after elective intracranial operations, the reasons for delayed extubation and mechanical ventilation mainly included large brain stem tumor resection, length of operation longer than six hours, and major intra-operative bleeding or brain swelling [19]. Mechanical ventilation was also continued in severe traumatic brain-injured patients after emergent operations. For patients after orthopedic and vascular surgery, delayed extubation was performed because of long duration of operation or major bleeding. Prolonged mechanical ventilation was usually required in patients with brain stem lesions and severe traumatic brain injury. In our unit, Pes monitoring was usually used in mechanically ventilated patients to guide ventilator settings and weaning. Exclusion criteria were as follows: 1) age under 18 years; 2) contraindications for esophageal balloon catheter insertion, including evidence of severe coagulopathy, diagnosed or suspected esophageal varices, and history of esophageal, gastric or lung surgery; and 3) evidence of active air leak from the lung, including bronchopleural fistula, pneumothorax, pneumomediastinum, and an existing chest tube. During the study, most of the patients did not recover from anesthesia and neuromuscular paralysis. In the case of recovered spontaneous breathing, continuous intravenous infusion of midazolam 0.05–0.2 mg/kg/h and fentanyl 0.1 mg/h were given, and an intravenous bolus of vecuronium 0.1 mg/kg was used as needed. The absence of spontaneous inspiratory effort was confirmed by the absence of a negative airway pressure (Paw) swing during a 3-s end-expiratory occlusion. The patients were ventilated under a volume-controlled mode with constant flow, set as the tidal volume of 6–8 ml/kg predicted body weight and clinical positive end-expiratory pressure and fraction of inspired oxygen. Hypoxemia was documented as the ratio of partial pressure of arterial oxygen to fraction of inspired oxygen less than 300. Acute respiratory distress syndrome (ARDS) was diagnosed according to the Berlin definition [20]. The absence of the chest X-ray criterion was defined as acute hypoxemic respiratory failure (AHRF).

The Cooper balloon was placed in the lower two thirds of the esophagus, which was confirmed by cardiac artifacts on Pes tracing and bedside X-ray examination.

During the study, the patients were remained in supine position with the head of the bed elevated to 30°. Before each balloon volume test, the balloon was inflated to 3.0 ml and then was deflated by generating a negative pressure followed by opening to the atmosphere for 3 min. The balloon was intermittently inflated in 0.2-ml increments up to 2.4 ml. At each tested volume, after 3-min equilibration, the airway was occluded at end-expiration and end-inspiration, each for 3 s. Positive pressure occlusion test was performed at end-expiratory occlusion, and the ratio of changes in Pes to Paw (∆Pes/∆Paw) during the compression of the chest wall was calculated [21].

Esophageal balloon pressure was plotted against the balloon volume (Fig. 2b). The method introduced by Mojoli et al. was used to determine the Ees and optimal balloon volume [13]. An intermediate linear section was visually inspected on end-expiratory balloon P-V curves, and the lower and upper limits were defined as the clinical V_MIN_ and V_MAX_. The range between V_MIN_ and V_MAX_ was defined as V_WORK_ and considered as the optimal balloon filling volume for clinical Pes measurement. The clinical V_BEST_ was defined as the balloon volume with the largest difference between end-expiratory and end-inspiratory Pes. The slope of the intermediate linear section on the end-expiratory balloon P-V curve was defined as the Ees [13]. Esophageal wall recoil pressure reacting to balloon filling; i.e., Pew, was estimated by the product of Ees and the balloon volume within V_MIN_ to V_MAX_, using the method previously introduced by Milic-Emili et al. [12]. The raw Pes values were calibrated by extrapolating to the zero balloon volume, which could also be expressed as: calibrated Pes = raw Pes - Pew.

In each of the patient’s balloon volume test, intermediate linear section on the end-expiratory balloon P-V curve (i.e. range of clinical V_MIN_ to V_MAX_) enclosed filling volume of 0.6 to 1.4 ml. Therefore, we simplified the estimation of Ees only using parameters at these two filling volumes, as the difference of end-expiratory balloon pressure between 0.6 ml and 1.4 ml divided by 0.8 ml (1.4–0.6 ml).

### Statistical analysis

Categorical variables are reported as numbers and percentages. Continuous data are presented as the median and interquartile range (IQR) and were compared using a Kruskal-Wallis test with post hoc comparison by Bonferroni’s correction.

Correlation of optimal balloon volume with balloon pressure was analyzed. Spearman’s correlation coefficient (rho) was calculated.

Bland-Altman’s analysis was used to examine the agreement for chamber elastance and baseline chamber pressure between the estimated and the measured value in the bench experiment, and for Ees between values calculated by the standard and the simple method in the clinical study. Bias and standard deviation (SD) of the mean bias were calculated. Upper and lower limits of agreement were defined as bias ±1.96 SD of the mean bias. The sample size in the bench experiment was based on the conditions’ setup (*n* = 30). The sample size in the clinical study (*n* = 40) was chosen on the basis of previous studies [13, 21]. The respective sample size gave a 95% confidence interval of the agreement of limit as ±0.32 × SD and ±0.27 × SD [22].

Analyses were conducted using SPSS V.20.0 (SPSS Inc., Chicago, IL, USA). A *p*-value < 0.05 was considered statistically significant.

## Results

### Bench experiment

Detailed experimental results are provided in Additional file 2. Example balloon P-V curves at atmospheric pressure and different levels of chamber elastance are shown in Additional file 3: Figure S1. The slope of the intermediate linear section on the balloon P-V curve at atmospheric pressure was 0.4 (0.3–0.4) cmH_2_O/ml. In accordance with previous investigations, gas-tight chambers with different inner volumes produced different levels of chamber elastance (Additional file 4: Table S1).

**Fig. 3:**
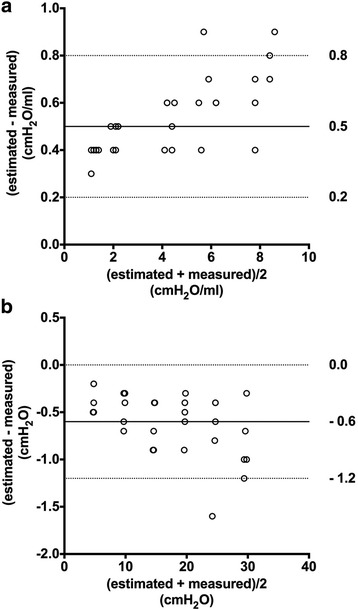
Bland-Altman’s limits of agreements analysis for estimated chamber elastance (panel **a**) and baseline chamber pressure (panel **b**). Horizontal axis represents the average of the estimated and measured values, and vertical axis represents the difference of estimated and measured values. Bias (solid line) and upper and lower limits of agreements (dash lines) are also shown

Bias (lower and upper limits of agreement) was 0.5 (0.2 to 0.8) cmH_2_O/ml between the slope of the linear section on the balloon P-V curve (estimated chamber elastance) and measured chamber elastance, and − 0.6 (− 1.2 to 0.0) cmH_2_O between the estimated and measured baseline chamber pressure (Fig. 3).

Optimal balloon volume parameters in the bench experiment are shown in Table 1. Bench V_MIN_ and V_MAX_ correlated linearly with balloon pressure (rho = 0.715 and 0.674, *p* = 0.010), but no correlation was found between V_BEST_ and balloon pressure (rho = 0.284, *p* = 0.128).

**Table 1:** Optimal balloon volume (ml) in the bench experiment and clinical study

	At atmosphere	In chambers	In patients
V_MIN_	all 0.2	0.2 (0.2–0.4) [0.2–0.4]	0.3 (0.2–0.4) [0.2–0.6]
V_MAX_	all 2.0	2.0 (2.0–2.2) [1.6–2.4]	1.6 (1.4–1.8) [1.4–2.2]
V_WORK_	all 1.8	1.8 (1.8–2.0) [1.4–2.0]	1.2 (1.1–1.4) [0.8–2.0]
V_BEST_	0.4 (0.4–0.6) [0.4–0.6]	0.4 (0.4–0.6) [0.4–0.8]	1.0 (0.8–1.2) [0.6–1.4]

*V*_*MIN*_ *and V*_*MAX*_: the minimal and maximal balloon volume representing the optimal filling volume with the least influence of balloon recoil pressure, *V*_*WORK*_: the difference between V_MIN_ and V_MAX_, *V*_*BEST*_ the best balloon filling volume

Data are shown as median (interquartile range) [range]

### Clinical study

Forty patients were enrolled. Baseline characteristics are shown in Table 2. Hypoxemia was presented in 13 (32.5%) patients, with ARDS and AHRF diagnosis in 4 (10%) and 9 (22.5%) patients, respectively. Detailed results in the clinical study are presented in Additional file 5.

**Table 2:** Baseline characteristics of the patients (*n* = 40)

Characteristic	Value
Age, years	43 (26–55)
Male, *n* (%)	27 (67.5)
Body mass index, kg/m^2^	24 (23–26)
APACHE II score	13 (12–15)
PaO_2_/FiO_2_	338 (210–373)
Tidal volume/PBW, ml/kg	6.8 (6.1–7.2)
PEEP, cmH_2_O	9 (6–11)
P_plat_, cmH_2_O	20.1 (18.3–22.4)
P_driv_, cmH_2_O	11.6 (9.7–12.4)
E_RS_, cmH_2_O/L	22.2 (17.5–25.2)
Reasons for delay extubation and MV, *n* (%)	
Large brain stem tumor resection	15 (37.5)
Severe traumatic brain injury	5 (12.5)
Long duration of operation in orthopedic surgery	15 (37.5)
Major bleeding in vascular surgery	5 (12.5)
Presence of hypoxemia, *n* (%)	
ARDS	4 (10.0)
AHRF	9 (22.5)

*AHRF* acute hypoxemic respiratory failure, *APACHE* Acute Physiology and Chronic Health Evaluation, *ARDS* acute respiratory distress syndrome, *E*_*RS*_ elastance of respiratory system, *MV* mechanical ventilation, *PaO*_*2*_*/FiO*_*2*_ ratio of partial pressure of arterial oxygen to fraction of inspired oxygen, *PBW* predicted body weight, *PEEP* positive end-expiratory pressure, *P*_*plat*_ airway plateau pressure, *P*_*driv*_ airway driving pressure

Continuous data are shown as median (interquartile range)

An intermediate linear section was visually identified on the end-expiratory esophageal balloon P-V curve in each of the clinical tests. Optimal balloon volume parameters obtained in the clinical study are also shown in Table 1 and Additional file 6: Table S2. V_MIN_ and V_MAX_ correlated linearly with either end-expiratory or end-inspiratory esophageal balloon pressures (rho = 0.320 to 0.352, *p* = 0.026 to 0.044). V_BEST_ was 1.0 (0.8–1.2) ml, ranging from 0.6 to 1.4 ml. No significant correlation was found between the V_BEST_ and esophageal balloon pressure (rho = 0.096 and 0.176, *p* = 0.557 and 0.279). Ees was 3.3 (2.5–4.1) cmH_2_O/ml, ranging from 1.2 to 5.8 cmH_2_O/ml. Estimated Pew at V_BEST_ was 2.8 (2.5–3.5) cmH_2_O, ranging from 1.4 to 5.2 cmH_2_O.

In vivo and in vitro curves closely resembled each other in the intermediate linear section, but in most cases, in vivo P-V curve elevated earlier and more significantly than the paired in vitro curve. Examples of in vivo esophageal balloon P-V curves during end-expiratory occlusion in combination with in vitro curves in the chambers with comparable elastance and baseline pressure are shown in Additional file 7: Figure S2.

In each of the patient’s tests, the V_MIN_ to V_MAX_ range enclosed 0.6 to 1.4 ml, and V_BEST_ was also located within 0.6 to 1.4 ml (Table 1). ∆Pes/∆Paw ratios during occlusion test were 0.725 (0.251–0.848) at balloon volumes below 0.6 ml, with 35.0% (42/120) within 0.8 to 1.2. Ratios at balloon volumes above 1.4 ml were 0.884 (0.734–1.041), and 59.4% (98/165) was within 0.8 to 1.2. At balloon volumes between 0.6 and 1.4 ml, there were 92.5% (185/200) ∆Pes/∆Paw ratios within 0.8 to 1.2, and no significant difference was found among different balloon volumes within this range (*p* = 0.067, Table 3). There was also no significant difference in the calibrated Pes (*p* = 0.997) among balloon volumes within 0.6 to 1.4 ml (Table 3).

**Table 3:** Calibrated esophageal pressure and occlusion test results at balloon volume between 0.6 and 1.4 ml in the clinical study

Volume	Calibrated Pes (cmH_2_O)^a^	∆Pes/∆Paw
0.6 ml	7.0 (4.2–9.8) [0.2–13.9]	0.900 (0.837–0.983) [0.592–1.235]
0.8 ml	6.7 (4.1–9.8) [0.6–13.9]	0.968 (0.861–1.004) [0.784–1.220]
1.0 ml	6.8 (3.8–9.8) [0.9–13.9]	0.974 (0.890–1.025) [0.809–1.180]
1.2 ml	6.8 (4.0–9.7) [0.1–13.8]	0.953 (0.887–1.068) [0.812–1.352]
1.4 ml	7.0 (4.4–9.8) [0.0–13.9]	0.972 (0.881–1.062) [0.800–1.494]
*p*	0.977	0.067

*Pes* Esophageal pressure, *∆Pes/∆Paw* the ratio of changes in esophageal pressure and airway pressure during positive pressure occlusion test by compression of the chest wall

Data are presented as median (interquartile range) [range] and were compared using Kruskal-Wallis test with post hoc comparison by Bonferroni correction

^a^Esophageal pressure was calibrated by the product of esophageal wall elastance and the balloon volume

Compared with the standard linear regression method, bias (lower and upper limits of agreement) of Ees estimated by the simple method was − 0.1 (− 0.7 to 0.6) cmH_2_O/ml (Additional file 8: Figure S3).

## Discussion

For the small-volume-balloon catheter investigated in the present study, we found that: 1) the slope of the intermediate linear section on the balloon P-V curve agreed with simulated balloon-surrounding elastance in our bench model; and 2) Ees estimated by the simple method agreed closely with the standard linear regression method.

It is well known that reliable Pes measurement depends on proper filling of the balloon [9, 10]. However, even within the optimal balloon volume range, esophageal wall reaction to balloon filling may elevate the absolute value of Pes [12, 13]. Milic-Emili et al. first proposed correction of raw Pes values by extrapolating to the zero balloon volume in healthy volunteers during spontaneous breathing [12]. Mojoli and colleagues further introduced a Pes calibration procedure by estimating the Ees and Pew in passive patients under controlled ventilation [13]. In the present bench experiment, glass chambers with different inner volumes were used to simulate different balloon-surrounding elastance (Fig. 1). The chamber elastance was also estimated using the slope of the intermediate linear section on the balloon P-V curve when the balloon was inflated in the chambers (Fig. 2a). Agreement analysis showed that the slope of the intermediate section on the balloon P-V curve overestimated chamber elastance by a mean bias of 0.5 cmH_2_O/ml (Fig. 3a), which approximated the slope of the linear section on the balloon P-V curve at atmospheric pressure (0.4 cmH_2_O/ml). While no surrounding elastance existed when the balloon was tested at atmosphere, we reasoned that a slight inclination of the linear section on the balloon P-V curve might have resulted from the tendency of the balloon returning to its original shape. We also found that intermediate sections on in vitro and in vivo balloon P-V curves were nearly overlaid (Additional file 7: Figure S2), additionally supporting the rationality of the methodology for Ees estimation.

Previous in vitro experiments found that V_WORK_, i.e., the balloon volume range between V_MIN_ and V_MAX_, was narrower in small-volume-balloons [9–11]. Among the tested esophageal catheter types, the geometric volume of the Cooper balloon was the smallest (2.8 ml). Thus, we questioned whether in vivo determination of Ees and calibration of Pes introduced by Mojoli et al. could be used in this type of balloon. Although our clinical V_WORK_ (median of 1.2 ml, ranging from 0.8 to 2.0 ml) was smaller than that in large-volume balloon (respective mean V_MIN_ and V_MAX_ of 1.5 and 5.4 ml) [13], an intermediate linear section on the end-expiratory P-V curve could be observed in each of the clinical balloon volume tests with at least five P-V data points for linear fitting (Fig. 2 and Additional file 7: Figure S2). This intermediate section is adequate for the calculation of Ees. In accordance with previously reported in vitro and in vivo studies [10, 13], V_MIN_ directly correlated with the balloon-surrounding pressure in our bench experiment and with Pes in our clinical study. However, we did not find a positive relationship between V_BEST_ with balloon pressure in either in vitro or in vivo condition. Additionally, Mojoli et al. found that the V_BEST_ for large-volume-balloon displayed significant variability in different bench conditions [10] and among patients [13], while in the present study the V_BEST_ was quiet similar among patients (Table 1). These results might be explained by the low variation in balloon with small geometric volume.

Our clinical results suggested that inflating the small-volume balloon also induced esophageal wall reactions. In our group of patients, the median Ees was 3.3 (2.5–4.1) cmH_2_O/ml, corresponding to a median Pew at V_BEST_ of 2.8 (2.5–3.5) cmH_2_O with the maximum value of 5.2 cmH_2_O. This level of Pew might be large enough to significantly overestimate the Pes and underestimate the transpulmonary pressure. Thus, we recommend that the balloon volume test and Pes calibration should be performed even when a small-volume balloon is used. Moreover, it could be noticed that in vivo V_BEST_ was markedly larger than in vitro value (Table 1), which might suggest the contact reaction of the balloon to the esophageal wall. However, this phenomenon requires further investigation.

Because the range of V_MIN_ to V_MAX_ was 0.6 to 1.4 ml in all clinical tests, we simplified the estimation of Ees by only using the esophageal balloon pressures measured at these two balloon volumes. The results obtained by the simple method agreed closely with those by the conventional linear regression method (Additional file 8: Figure S3). For the small-volume balloon used in the present study, all clinical V_BEST_ were also located between 0.6 and 1.4 ml, and no significant difference was found in either the ∆Pes/∆Paw ratio during the occlusion test or calibrated Pes among balloon volumes within this range (Table 3). Therefore, we further suggest a simple procedure for balloon volume test and Pes calibration for the Cooper balloon catheter. The balloon can only be inflated to three volumes of 0.6, 1.0 and 1.4 ml, and the volume with largest tidal swing in balloon pressure could be selected as the V_BEST_. Ees can be simply estimated only using pressures at balloon volumes of 0.6 and 1.4 ml, and the raw Pes at the V_BEST_ can be calibrated. Whether this procedure is suitable for other types of balloons, especially for large volume balloons, needs further investigation.

The major strength of our study is the combined reporting of bench and clinical results. There are limitations in our study. First, although different levels of balloon-surrounding elastance were successfully produced in our bench experiment, we definitely recognized that our model did not simulate the real scenario within the esophagus because the esophageal contact reaction to the balloon inflation was omitted. This limitation can be illustrated in the merged in vitro and in vivo balloon P-V curves (Additional file 7: Figure S2). At higher balloon volumes, in vivo curves elevated earlier and more markedly, which might be due to the esophageal contact reaction. However, our simulated levels of balloon-surrounding elastance and pressure covered clinical reported ranges of Ees and Pes [3, 4, 6, 7, 13, 21, 23]. When combining the results in the bench experiment and the clinical study, we reasonably thought that this simplified bench model was enough to demonstrate the relationship between the slope of the intermediate balloon P-V curve and the Ees. Second, we only investigated one specific type of balloon with a relatively small geometric volume. Third, only postoperative patients with normal oxygenation and airway driving pressure were studied. Fourth, balloon volume test was only performed in passive patients in controlled ventilation. Therefore, our findings cannot be directly generalized to other balloon catheters and other patient populations.

## Conclusions

Using a bench model composed of different gas-tight chambers with different inner volumes, we assessed the relationship between the slope of the intermediate linear section on the balloon P-V curve and balloon-surrounding elastance. Methods of Ees estimation and Pes calibration were also applicable for a balloon with a relatively small geometric volume. For the Cooper balloon catheter, a simple bedside procedure for the selection of the optimal balloon volume and the calibration of Pes can be used. Further investigations are needed to clarify our results in other types of balloon catheters and patient populations.

## Additional files

Additional file 1: Detailed methods in the bench experiment and clinical study. (PDF 110 kb)

Additional file 2: Detailed results in the bench experiment. (PDF 110 kb)

Additional file 3: **Figure S1.** Example balloon P-V curves at atmospheric pressure and different levels of chamber elastance. (PDF 468 kb)

Additional file 4: **Table S1.** Measured chamber elastance (cmH_2_O/ml) in chambers with different inner volumes. (PDF 62 kb)

Additional file 5: Detailed results in the clinical study (PDF 172 kb)

Additional file 6: **Table S2.** Individual data of balloon volume and esophageal wall elastance. (PDF 71 kb)

Additional file 7: **Figure S2.** Examples of in vivo esophageal balloon pressure-volume curve during end-expiratory occlusion in combination with in vitro curve in chamber with comparable elastance and baseline pressure. (PDF 571 kb)

Additional file 8: **Figure S3.** Bland-Altman’s limits of agreements analysis for the estimated esophageal elastance by the simple and standard linear regression method. (PDF 287 kb)

## Abbreviations

∆Pes/∆Paw: Ratio of changes in esophageal pressure and airway pressure positive pressure occlusion test;
AHRF: Acute hypoxemic respiratory failure
ARDS: Acute respiratory distress syndrome
Ees: Esophageal wall elastance
IQR: Interquartile range
MV: Mechanical ventilation
Paw: Airway pressure
Pes: Esophageal pressure
Pew: Pressure generated by the esophageal wall during balloon inflation
P-V curve: Pressure-volume curve
V_BEST_: The best balloon filling volume
V_MIN_ and V_MAX_: The minimal and maximal balloon volumes representing the optimal filling volume with the least influence of balloon recoil pressure
V_WORK_: The difference between V_MIN_ and V_MAX_
