# Supplementary material for: Use of esophageal balloon pressure-volume curve analysis to determine esophageal wall elastance and calibrate raw esophageal pressure: a bench experiment and clinical study
Source: BMC Anesthesiol. 2018 Feb 14;18:21. doi: 10.1186/s12871-018-0488-6 (PMC5813414; doi:10.1186/s12871-018-0488-6)
Supplement: Supplementary file 1 — Detailed methods in the bench experiment and clinical study. (PDF 110 kb) [file 12871_2018_488_MOESM1_ESM.pdf]

Use of esophageal balloon pressure-volume curve analysis to determine esophageal wall elastance and calibrate raw esophageal pressure: a bench experiment and clinical study

Xiu-Mei Sun, Guang-Qiang Chen, Hua-Wei Huang, Xuan He, Yan-Lin Yang,

Zhong-Hua Shi, Ming Xu, Jian-Xin Zhou

Additional file 1: Detailed methods

Esophageal balloon catheter

In the present study, we used a commercially available esophageal balloon catheter (Cooper: LOT 177405, Cooper Surgical, USA) of 5 Fr in diameter and 85 cm in length, enclosed with a polyethylene balloon of 2.8 ml in geometric volume [S1]. The balloon filling volume is recommended as 1.0 ml by the manufacturer [S2, S3].

Pressure measurements

During the study, pressures were measured by pressure transducers (KT 100D-2, Kleis TEK, Italy, range: +/- 100 cmH_2O) connected to an ICU-Lab Pressure Box (ICU Lab, KleisTEK Engineering, Bari, Italy) by 80 cm rigid tube lines [S1]. Signals were displayed continuously and saved at a sample rate of 200 Hz in a laptop computer for further analysis (ICU-Lab 2.5 Software Package, ICU Lab, KleisTEK Engineering, Bari, Italy). Pressure transducers were pre-calibrated using two points calibration function in the ICU-Lab, with one reference as atmospheric pressure and another one as 10 cm water column in a U shape tube.

Bench experiment

The bench experiment was conducted in an 8-m^2 laboratory. The room temperature was maintained at 25 °C by an air conditioner during the experiment.

Glass chambers were used to simulate different balloon-surrounding conditions. Each chamber had two openings: one for introducing the balloon into the chamber and the other one for adjusting and measuring the chamber pressure. Gas-tight glass chambers with different inner volumes contain different amounts of air. According to Boyle's law and relative molecular mass, when injecting additional volumes of air, different elastance conditions can be produced in different chambers with different inner volumes [S2, S4]. We used five glass chambers with an inner volume of 1000, 500, 250, 175 and 125 ml to obtain five levels of chamber elastance approximately from 1 to 8 cmH_2O/ml. This range of elastance covered reported human Ees [S5, S6]. In each chamber, six levels of baseline chamber pressure (5, 10, 15, 20, 25 and 30 cmH_2O) were also established by injecting different amounts of air into the chamber. This range of pressure covered reported Pes in passive patients under controlled ventilation [S7-S11]. Thus, 30 balloon-surrounding conditions with five levels of chamber elastance and six levels of baseline chamber pressure were simulated.

Five Cooper catheter packages were selected. After being unpacked, the balloon was inflated to 3.0 ml (slightly above the geometric volume) and visually inspected to exclude leaks. The five balloon catheters were randomly introduced into the five different chambers, and the balloon lumen of the catheter was secured with a 3-way

stopcock. During the experiment, connections in the chamber system were sealed with silicone sealant. After a positive pressure of 30 cmH_2O was added in the chamber, systematic leaks were excluded if decreasing in the chamber pressure was less than 1 cmH_2O during the first 1-min equilibrating period.

Before each balloon volume test, the residual volume of the balloon was standardized by adding a positive pressure surrounding the balloon [S12-S14]. Briefly, the chamber pressure was adjusted to 5 cmH_2O by air injection. The balloon was inflated to 3.0 ml and then was deflated by generating negative pressure followed by opening to the atmosphere. After 3-min equilibration, the 3-way stopcock was closed. We arbitrarily defined the balloon volume under this condition as the zero filling volume [S12-S14]. The balloon was intermittently inflated in 0.2-ml increments up to 2.4 ml, using a 1.0-ml gas-tight syringe (LOT JM00B25, Runze fluid control equipment, C.O., Ltd., Nanjing, China). A balloon volume test was first performed at atmospheric pressure and then in each chamber with certain elastance at different levels of baseline chamber pressure.

Balloon pressure and chamber pressure were simultaneously measured. Balloon transmural pressure was defined as the difference between the balloon pressure and the chamber pressure (balloon pressure - chamber pressure) [S2, S3]. Balloon transmural pressure at atmospheric pressure was equal to the balloon pressure. The balloon volume with transmural pressure within ± 1.0 cmH_2O was defined as the minimal and maximal balloon volumes (V_{MIN} and V_{MAX}), which represented the optimal filling volume with the least influence of balloon recoil pressure [S2, S3].

Balloon working volume (V_{WORK}) was calculated as the difference between V_{MIN} and V_{MAX}. The balloon volume with the closest to zero transmural pressure was defined as the best filling volume (V_{BEST}) in the bench experiment.

Balloon pressure and chamber pressure were plotted against the balloon volume. The chamber pressure increased linearly as a function of balloon volume, and the slope obtained by least square fitting was defined as the measured chamber elastance. The balloon P-V curve exhibited a sigmoid shape with an intermediate linear section corresponding to the optimal filling volume range (V_{MIN} to V_{MAX}). We used the slope of this linear section to estimate chamber elastance. Baseline chamber pressure was estimated as the difference between the measured balloon pressure at V_{BEST} and the product of estimated chamber elastance and V_{BEST}.

Clinical study

The clinical study was conducted in the intensive care unit, Beijing Tiantan Hospital, Capital Medical University, Beijing, China. The study protocol was reviewed and approved by the local Institutional Review Board (KY-2016-11-22) and the study was registered at ClinicalTrials.gov (NCT02976844). Written informed consent was obtained from the patient or appropriate substitute decision makers. We enrolled postoperative patients with delayed emergence from general anesthesia admitted to the unit for mechanical ventilation. In our unit, esophageal pressure (Pes) monitoring was usually performed in these patients to guide ventilator settings and weaning. Exclusion criteria were as follows: 1) age under 18 years; 2) contraindications for

esophageal balloon catheter insertion, including evidence of severe coagulopathy, diagnosed or suspected esophageal varices, and history of esophageal, gastric or lung surgery; and 3) evidence of active air leak from the lung, including bronchopleural fistula, pneumothorax, pneumomediastinum, and an existing chest tube. During the study, most of the patients did not recover from anesthesia and neuromuscular paralysis. In the case of recovered spontaneous breathing, continuous intravenous infusion of midazolam 0.05–0.2 mg/kg/h and fentanyl 0.1 mg/h were given, and an intravenous bolus of vecuronium 0.1 mg/kg was used as needed. The absence of spontaneous inspiratory effort was confirmed by the absence of a negative airway pressure (Paw) swing during a 3-second end-expiratory occlusion. The patients were ventilated under a volume-controlled mode with constant flow, set as the tidal volume of 6-8 ml/kg predicted body weight and clinical positive end-expiratory pressure and fraction of inspired oxygen. The ICU physician accompanied the patient and ensured the patient's safety.

Cooper catheter was also used in the clinical study. The balloon was placed in the lower two thirds of the esophagus, which was confirmed by cardiac artifacts on Pes tracing and bedside X-ray examination.

Before each balloon volume test, the balloon was inflated to 3.0 ml and then was deflated by generating a negative pressure followed by opening to the atmosphere for 3 min. The balloon was intermittently inflated in 0.2-ml increments up to 2.4 ml. In some patients, the test was stopped when the balloon was inflated by 1.8 to 2.2 ml because of marked elevation of esophageal balloon pressure. At each tested balloon

volume, after 3 min equilibration, the airway was occluded at end-expiration and end-inspiration, each for 3 s. Positive pressure occlusion test was performed at end-expiratory occlusion, and the ratio of changes in Pes to airway pressure (ΔPes/ΔPaw) during the compression of the chest wall was calculated [S11]. Esophageal balloon pressure was plotted against the balloon volume. The method introduced by Mojoli et al. was used to determine the Ees and optimal balloon volume [S5]. An intermediate linear section was visually inspected on end-expiratory balloon P-V curves, and the lower and upper limits were defined as the clinical V_{MIN} and V_{MAX}. The range between V_{MIN} and V_{MAX} was defined as V_{WORK} and considered as the optimal balloon filling volume for clinical Pes measurement. The clinical V_{BEST} was defined as the balloon volume with the largest difference between end-expiratory and end-inspiratory Pes. The slope of the intermediate linear section on the end-expiratory balloon P-V curve was defined as the Ees [S5]. Esophageal wall recoil pressure reacting to balloon filling; i.e., Pew, was estimated by the product of Ees and the balloon volume within V_{MIN} to V_{MAX}, which could be used to calibrate the raw Pes [S15].

Because balloon volumes between 0.6 and 1.4 ml were located within the range of clinical V_{MIN} to V_{MAX} in all patients' tests, we further simplified the estimation of Ees as the difference of end-expiratory balloon pressure at 0.6 ml and 1.4 ml divided by 0.8 ml.

Statistical analysis

Categorical variables are reported as numbers and percentages. Continuous data are presented as the median and interquartile range (IQR) and were compared using a Kruskal-Wallis test with post hoc comparison by Bonferroni's correction. Correlation of optimal balloon volume with balloon pressure was analyzed. Spearman's correlation coefficient (rho) was calculated.

Bland-Altman's analysis was used to examine the agreement for chamber elastance and baseline chamber pressure between the estimated and the measured value in the bench experiment, and for Ees between values calculated by standard and simple methods in the clinical study. Bias and standard deviation (SD) of the mean bias were calculated. Upper and lower limits of agreement were defined as bias ± 1.96 SD of the mean bias. The sample size in the bench experiment was based on the conditions' setup. The sample size in the clinical study was chosen on the basis of previous studies [S5, S11]. The respective sample size gave a 95 % confidence interval of the agreement of limit as ± 0.32×SD and ± 0.27×SD [S16].

References

S1. Yang YL, He X, Sun XM, Chen H, Shi ZH, Xu M, et al. Optimal esophageal balloon volume for accurate estimation of pleural pressure at end-expiration and end-inspiration: an in vitro bench experiment. Intensive Care Med Exp. 2017;5:35.

S2. Walterspacher S, Isaak L, Guttmann J, Kabitz HJ, Schumann S. Assessing respiratory function depends on mechanical characteristics of balloon catheters.

Respir Care. 2014;59:1345-52.

S3. Mojoli F, Chiumello D, Pozzi M, Algieri I, Bianzina S, Luoni S, et al. Esophageal pressure measurements under different conditions of intrathoracic pressure. An in vitro study of second generation balloon catheters. Minerva Anestesiol. 2015;81:855-64.

S4. Haberthür C, Mehlig A, Stover JF, Schumann S, Möller K, Priebe HJ, et al. Expiratory automatic endotracheal tube compensation reduces dynamic hyperinflation in a physical lung model. Crit Care. 2009;13:R4.

S5. Mojoli F, Iotti GA, Torriglia F, Pozzi M, Volta CA, Bianzina S, et al. In vivo calibration of esophageal pressure in the mechanically ventilated patient makes measurements reliable. Crit Care. 2016;20:98.

S6. Hedenstierna G, Järnberg PO, Torsell L, Gottlieb I. Esophageal elastance in anesthetized humans. J Appl Physiol Respir Environ Exerc Physiol. 1983;54:1374-8.

S7. Talmor D, Sarge T, O'Donnell CR, Ritz R, Malhotra A, Lisbon A, et al. Esophageal and transpulmonary pressures in acute respiratory failure. Crit Care Med. 2006;34:1389-94.

S8. Talmor D, Sarge T, Malhotra A, O'Donnell CR, Ritz R, Lisbon A, et al. Mechanical ventilation guided by esophageal pressure in acute lung injury. N Engl J Med. 2008;359:2095-104.

S9. Loring SH, O'Donnell CR, Behazin N, Malhotra A, Sarge T, Ritz R, et al. Esophageal pressures in acute lung injury: do they represent artifact or useful

information about transpulmonary pressure, chest wall mechanics, and lung stress. J Appl Physiol. 2010;108:515-22.

S10. Chen L, Chen GQ, Shore K, Shklar O, Martins C, Devenyi B, et al. Implementing a bedside assessment of respiratory mechanics in patients with acute respiratory distress syndrome. Crit Care. 2017;21:84.

S11. Chiumello D, Consonni D, Coppola S, Froio S, Crimella F, Colombo A. The occlusion tests and end-expiratory esophageal pressure: measurements and comparison in controlled and assisted ventilation. Ann Intensive Care.2016;6:13.

S12. Lemen R, Benson M, Jones JG. Absolute pressure measurements with hand-dipped and manufactured esophageal balloons. J Appl Physiol. 1974;37:600-3.

S13. Beardsmore CS, Helms P, Stocks J, Hatch DJ, Silverman M. Improved esophageal balloon technique for use in infants. J Appl Physiol Respir Environ Exerc Physiol. 1980;49:735-42.

S14. Cross TJ, Lalande S, Hyatt RE, Johnson BD. Response characteristics of esophageal balloon catheters handmade using latex and nonlatex materials. Physiol Rep. 2015;3:e12426.

S15. Milic-Emili J, Mead J, Turner JM, Glauser EM. Improved technique for estimating pleural pressure from esophageal balloons. J Appl Physiol. 1964;19:207-11.

S16. Bland JM, Altman DG. Statistical methods for assessing agreement between two methods of clinical measurement. Lancet. 1986;1(8476):307-10.
