# Supplementary material for: Use of esophageal balloon pressure-volume curve analysis to determine esophageal wall elastance and calibrate raw esophageal pressure: a bench experiment and clinical study
Source: BMC Anesthesiol. 2018 Feb 14;18:21. doi: 10.1186/s12871-018-0488-6 (PMC5813414; doi:10.1186/s12871-018-0488-6)
Supplement: Supplementary file 2 — Detailed results in the bench experiment. (PDF 110 kb) [file 12871_2018_488_MOESM2_ESM.pdf]

Use of esophageal balloon pressure-volume curve analysis to determine esophageal wall elastance and calibrate raw esophageal pressure: a bench experiment and clinical study

Xiu-Mei Sun, Guang-Qiang Chen, Hua-Wei Huang, Xuan He, Yan-Lin Yang, Zhong-Hua Shi, Ming Xu, Jian-Xin Zhou

Additional file 2: Detailed results in the bench experiment

Balloon volume tests were performed at atmospheric pressure and in chambers with different inner volumes at different baseline pressures. The balloon was intermittently inflated in 0.2-ml increment up to 2.4 ml. Balloon pressure and chamber pressure were simultaneously measured. Balloon transmural pressure was defined as the difference between the balloon pressure and the chamber pressure (balloon pressure - chamber pressure). Balloon transmural pressure at atmospheric pressure was equal to the balloon pressure. The balloon volume range with transmural pressure within ± 1.0 cmH_2O was defined as the optimal balloon volume, which represented the filling volume with minimal influence of balloon recoil pressure.

Catheter No 1

		Balloon volume (ml)												
		0	0.2	0.4	0.6	0.8	1	1.2	1.4	1.6	1.8	2	2.2	2.4
**At atmosphere**	**Balloon pressure (cmH_2O)**	-3.064	-0.337	-0.041	0.057	0.080	0.096	0.126	0.156	0.190	0.319	0.651	1.493	5.698
**Chamber inner volume = 1000 ml**														
**Baseline pressure = 5 cmH_2O**	**Chamber pressure (cmH_2O)**	5.054	5.216	5.418	5.628	5.839	6.003	6.222	6.429	6.639	6.854	7.083	7.303	7.487
	**Balloon pressure (cmH_2O)**	0.415	4.997	5.378	5.637	5.894	6.094	6.356	6.616	6.911	7.299	7.935	9.087	12.349
**Baseline pressure = 10 cmH_2O**	**Chamber pressure (cmH_2O)**	9.861	10.011	10.208	10.436	10.628	10.856	11.046	11.274	11.497	11.710	11.913	12.109	12.290
	**Balloon pressure (cmH_2O)**	1.341	9.761	10.202	10.467	10.686	10.955	11.179	11.453	11.749	12.085	12.529	13.325	15.165
**Baseline pressure = 15 cmH_2O**	**Chamber pressure (cmH_2O)**	14.944	15.088	15.314	15.554	15.804	16.011	16.245	16.505	16.748	16.977	17.169	17.400	17.618
	**Balloon pressure (cmH_2O)**	6.546	14.792	15.297	15.585	15.869	16.131	16.410	16.724	17.037	17.399	17.835	18.631	20.231
**Baseline pressure = 20 cmH_2O**	**Chamber pressure (cmH_2O)**	19.947	20.065	20.084	20.484	20.697	20.920	21.130	21.337	21.567	21.779	21.978	22.180	22.393
	**Balloon pressure (cmH_2O)**	6.617	19.543	20.301	20.547	20.801	21.051	21.304	21.547	21.828	22.124	22.450	22.993	23.866
**Baseline pressure = 25 cmH_2O**	**Chamber pressure (cmH_2O)**	24.919	24.955	25.105	25.307	25.497	25.692	25.869	26.065	26.220	26.416	26.609	26.777	26.938
	**Balloon pressure (cmH_2O)**	1.572	23.124	24.983	25.339	25.584	25.816	26.015	26.229	26.407	26.633	26.906	27.246	27.752
**Baseline pressure = 30 cmH_2O**	**Chamber pressure (cmH_2O)**	29.933	29.986	30.149	30.346	30.529	30.723	30.912	31.113	31.304	31.490	31.666	31.846	32.042
	**Balloon pressure (cmH_2O)**	9.270	28.681	30.041	30.393	30.664	30.884	31.090	31.315	31.526	31.762	32.007	32.420	33.054

Catheter No 2

		Balloon volume (ml)												
		0	0.2	0.4	0.6	0.8	1	1.2	1.4	1.6	1.8	2	2.2	2.4
**At atmosphere**	**Balloon pressure (cmH_2O)**	-2.978	-0.205	-0.014	0.047	0.073	0.084	0.092	0.127	0.182	0.332	0.624	1.447	6.709
**Chamber inner volume = 500 ml**														
**Baseline pressure = 5 cmH_2O**	**Chamber pressure (cmH_2O)**	4.944	5.271	5.643	6.047	6.418	6.799	7.165	7.583	7.924	8.215	8.574	8.932	9.241
	**Balloon pressure (cmH_2O)**	1.629	5.071	5.618	6.159	6.562	6.973	7.370	7.827	8.253	8.674	9.368	10.598	12.861
**Baseline pressure = 10 cmH_2O**	**Chamber pressure (cmH_2O)**	10.054	10.349	10.756	11.143	11.547	11.954	12.363	12.811	13.239	13.648	14.016	14.367	14.739
	**Balloon pressure (cmH_2O)**	2.387	9.998	10.782	11.270	11.714	12.158	12.600	13.074	13.568	14.095	14.711	15.683	17.756
**Baseline pressure = 15 cmH_2O**	**Chamber pressure (cmH_2O)**	15.010	15.231	15.648	16.057	16.449	16.867	17.264	17.647	18.062	18.470	18.882	19.274	19.635
	**Balloon pressure (cmH_2O)**	4.572	14.801	15.681	16.209	16.640	17.092	17.512	17.912	18.378	18.931	19.633	20.834	23.388
**Baseline pressure = 20 cmH_2O**	**Chamber pressure (cmH_2O)**	19.996	20.179	20.570	20.976	21.386	21.776	22.157	22.556	22.909	23.184	23.542	23.937	24.303
	**Balloon pressure (cmH_2O)**	5.098	19.474	20.585	21.119	21.574	21.994	22.400	22.829	23.216	23.552	24.002	24.713	25.921
**Baseline pressure = 25 cmH_2O**	**Chamber pressure (cmH_2O)**	24.939	25.028	25.420	25.807	26.193	26.586	26.986	27.396	27.802	28.206	28.596	29.009	29.387
	**Balloon pressure (cmH_2O)**	4.728	23.218	25.413	25.958	26.406	26.837	27.263	27.696	28.124	28.583	29.100	29.823	30.949
**Baseline pressure = 30 cmH_2O**	**Chamber pressure (cmH_2O)**	30.276	30.354	30.616	30.868	31.139	31.418	31.769	32.145	32.552	32.936	33.349	33.728	34.107
	**Balloon pressure (cmH_2O)**	6.889	26.475	30.071	30.683	31.110	31.482	31.872	32.285	32.728	33.154	33.602	34.063	34.633

Catheter No 3

		Balloon volume (ml)												
		0	0.2	0.4	0.6	0.8	1	1.2	1.4	1.6	1.8	2	2.2	2.4
**At atmosphere**	**Balloon pressure (cmH_2O)**	-3.053	-0.374	-0.090	0.003	0.033	0.054	0.060	0.077	0.135	0.269	0.590	1.436	7.302
**Chamber inner volume = 250 ml**														
**Baseline pressure = 5 cmH_2O**	**Chamber pressure (cmH_2O)**	4.937	5.623	6.439	7.321	8.162	9.017	9.849	10.708	11.550	12.389	13.209	14.019	14.765
	**Balloon pressure (cmH_2O)**	1.352	5.483	6.532	7.581	8.531	9.446	10.310	11.146	12.059	13.011	14.207	15.934	18.974
**Baseline pressure = 10 cmH_2O**	**Chamber pressure (cmH_2O)**	10.053	10.682	11.547	12.384	13.254	14.106	14.943	15.793	16.627	17.462	18.285	19.103	19.858
	**Balloon pressure (cmH_2O)**	1.502	10.290	11.637	12.526	13.427	14.304	15.204	16.094	17.000	17.929	18.970	20.204	21.970
**Baseline pressure = 15 cmH_2O**	**Chamber pressure (cmH_2O)**	14.917	15.420	16.220	17.090	17.946	18.796	19.646	20.508	21.349	22.204	23.025	23.858	24.627
	**Balloon pressure (cmH_2O)**	4.085	14.837	16.260	17.228	18.132	19.033	19.947	20.865	21.747	22.681	23.661	24.812	26.368
**Baseline pressure = 20 cmH_2O**	**Chamber pressure (cmH_2O)**	20.003	20.485	21.201	21.986	22.785	23.599	24.382	25.193	25.994	26.812	27.627	28.435	29.206
	**Balloon pressure (cmH_2O)**	8.543	19.602	21.137	22.022	22.866	23.688	24.557	25.416	26.284	27.198	28.162	29.239	30.609
**Baseline pressure = 25 cmH_2O**	**Chamber pressure (cmH_2O)**	25.000	25.315	26.130	26.974	27.771	28.560	29.335	30.145	30.924	31.712	32.471	33.241	33.994
	**Balloon pressure (cmH_2O)**	8.400	24.527	26.277	27.161	27.996	28.813	29.639	30.471	31.309	32.172	33.025	33.982	35.117
**Baseline pressure = 30 cmH_2O**	**Chamber pressure (cmH_2O)**	29.975	30.184	30.918	31.700	32.477	33.281	34.102	34.918	35.775	36.544	37.331	38.109	38.889
	**Balloon pressure (cmH_2O)**	8.453	28.045	31.044	31.918	32.728	33.537	34.398	35.238	36.145	36.975	37.846	38.766	39.498

Catheter No 4

		Balloon volume (ml)												
		0	0.2	0.4	0.6	0.8	1	1.2	1.4	1.6	1.8	2	2.2	2.4
**At atmosphere**	**Balloon pressure (cmH_2O)**	-3.006	-0.168	0.001	0.037	0.056	0.059	0.077	0.102	0.171	0.344	0.653	1.883	9.501
**Chamber inner volume = 175 ml**														
**Baseline pressure = 5 cmH_2O**	**Chamber pressure (cmH_2O)**	5.004	6.040	7.209	8.405	9.603	10.787	11.974	13.169	14.365	15.515	16.660	17.814	18.828
	**Balloon pressure (cmH_2O)**	0.852	5.558	7.093	8.416	9.734	10.992	12.271	13.499	14.741	16.021	17.453	19.360	22.292
**Baseline pressure = 10 cmH_2O**	**Chamber pressure (cmH_2O)**	9.974	10.906	12.132	13.275	14.475	15.666	16.856	18.059	19.234	20.445	21.619	22.742	23.797
	**Balloon pressure (cmH_2O)**	2.370	10.320	12.032	13.309	14.593	15.869	17.125	18.351	19.573	20.900	22.330	24.012	26.595
**Baseline pressure = 15 cmH_2O**	**Chamber pressure (cmH_2O)**	15.023	15.727	16.819	18.005	19.120	20.284	21.445	22.607	23.738	24.875	26.017	27.008	27.896
	**Balloon pressure (cmH_2O)**	2.739	14.987	16.635	18.013	19.217	20.467	21.697	22.906	24.073	25.312	26.605	27.933	29.357
**Baseline pressure = 20 cmH_2O**	**Chamber pressure (cmH_2O)**	19.973	20.525	21.635	22.797	23.943	25.121	26.281	27.440	28.750	29.424	30.434	31.344	32.517
	**Balloon pressure (cmH_2O)**	4.236	18.268	21.463	22.795	24.063	25.352	26.569	27.775	28.956	29.810	30.912	31.952	33.473
**Baseline pressure = 25 cmH_2O**	**Chamber pressure (cmH_2O)**	25.007	25.242	25.887	27.041	28.211	29.370	30.499	31.621	32.724	33.848	34.919	36.058	36.442
	**Balloon pressure (cmH_2O)**	5.664	22.088	25.468	26.957	28.242	29.490	30.713	31.928	33.082	34.246	35.403	36.715	37.718
**Baseline pressure = 30 cmH_2O**	**Chamber pressure (cmH_2O)**	30.056	30.281	30.918	32.288	33.352	34.435	35.542	36.584	37.663	38.754	39.844	40.912	41.922
	**Balloon pressure (cmH_2O)**	7.406	27.467	31.044	32.350	33.529	34.697	35.928	37.067	38.210	39.352	40.537	41.582	42.909

Catheter No 5

		Balloon volume (ml)												
		0	0.2	0.4	0.6	0.8	1	1.2	1.4	1.6	1.8	2	2.2	2.4
**At atmosphere**	**Balloon pressure (cmH_2O)**	-3.089	-0.251	-0.046	0.009	0.034	0.048	0.078	0.097	0.138	0.300	0.641	1.825	9.271
**Chamber inner volume = 125 ml**														
**Baseline pressure = 5 cmH_2O**	**Chamber pressure (cmH_2O)**	4.963	6.447	8.132	9.784	11.514	13.213	14.851	16.469	18.060	19.674	21.276	22.840	24.331
	**Balloon pressure (cmH_2O)**	0.443	6.128	8.248	10.082	11.872	13.555	15.302	17.167	19.008	20.999	23.283	25.872	29.123
**Baseline pressure = 10 cmH_2O**	**Chamber pressure (cmH_2O)**	10.063	11.208	12.844	14.442	16.109	17.758	19.418	21.032	22.637	24.303	25.929	27.505	28.982
	**Balloon pressure (cmH_2O)**	0.575	10.947	12.973	14.696	16.461	18.084	19.755	21.486	23.309	25.211	27.264	29.447	31.852
**Baseline pressure = 15 cmH_2O**	**Chamber pressure (cmH_2O)**	15.008	15.884	17.494	19.206	20.855	22.518	24.142	25.749	27.365	29.032	30.622	32.190	33.742
	**Balloon pressure (cmH_2O)**	1.900	15.450	17.611	19.474	21.211	22.869	24.484	26.195	27.963	29.914	31.904	34.074	36.463
**Baseline pressure = 20 cmH_2O**	**Chamber pressure (cmH_2O)**	20.008	20.692	22.266	23.831	25.466	27.048	28.688	30.242	31.791	33.312	34.932	36.458	37.949
	**Balloon pressure (cmH_2O)**	3.624	19.946	22.353	24.079	25.827	27.432	29.057	30.717	32.363	34.096	35.963	38.054	40.346
**Baseline pressure = 25 cmH_2O**	**Chamber pressure (cmH_2O)**	25.031	25.586	27.105	28.708	30.325	31.889	33.484	35.060	36.292	37.865	39.447	40.988	42.493
	**Balloon pressure (cmH_2O)**	5.472	23.504	27.103	28.947	30.699	32.317	33.839	35.454	36.754	38.506	40.356	42.262	44.153
**Baseline pressure = 30 cmH_2O**	**Chamber pressure (cmH_2O)**	29.865	30.306	31.707	33.324	34.925	36.571	38.132	39.718	41.309	42.448	43.973	45.506	47.010
	**Balloon pressure (cmH_2O)**	6.840	26.794	31.574	33.522	35.279	36.996	38.536	40.126	41.830	43.055	44.828	46.644	48.591
