# Supplementary material for: Use of esophageal balloon pressure-volume curve analysis to determine esophageal wall elastance and calibrate raw esophageal pressure: a bench experiment and clinical study
Source: BMC Anesthesiol. 2018 Feb 14;18:21. doi: 10.1186/s12871-018-0488-6 (PMC5813414; doi:10.1186/s12871-018-0488-6)
Supplement: Supplementary file 3 — Figure S1. Example balloon P-V curves at atmospheric pressure and different levels of chamber elastance. (PDF 468 kb) [file 12871_2018_488_MOESM3_ESM.pdf]

Use of esophageal balloon pressure-volume curve analysis to determine esophageal wall elastance and calibrate raw esophageal pressure: a bench experiment and clinical study

Xiu-Mei Sun, Guang-Qiang Chen, Hua-Wei Huang, Xuan He, Yan-Lin Yang,

Zhong-Hua Shi, Ming Xu, Jian-Xin Zhou

Additional file 3: Figure S1

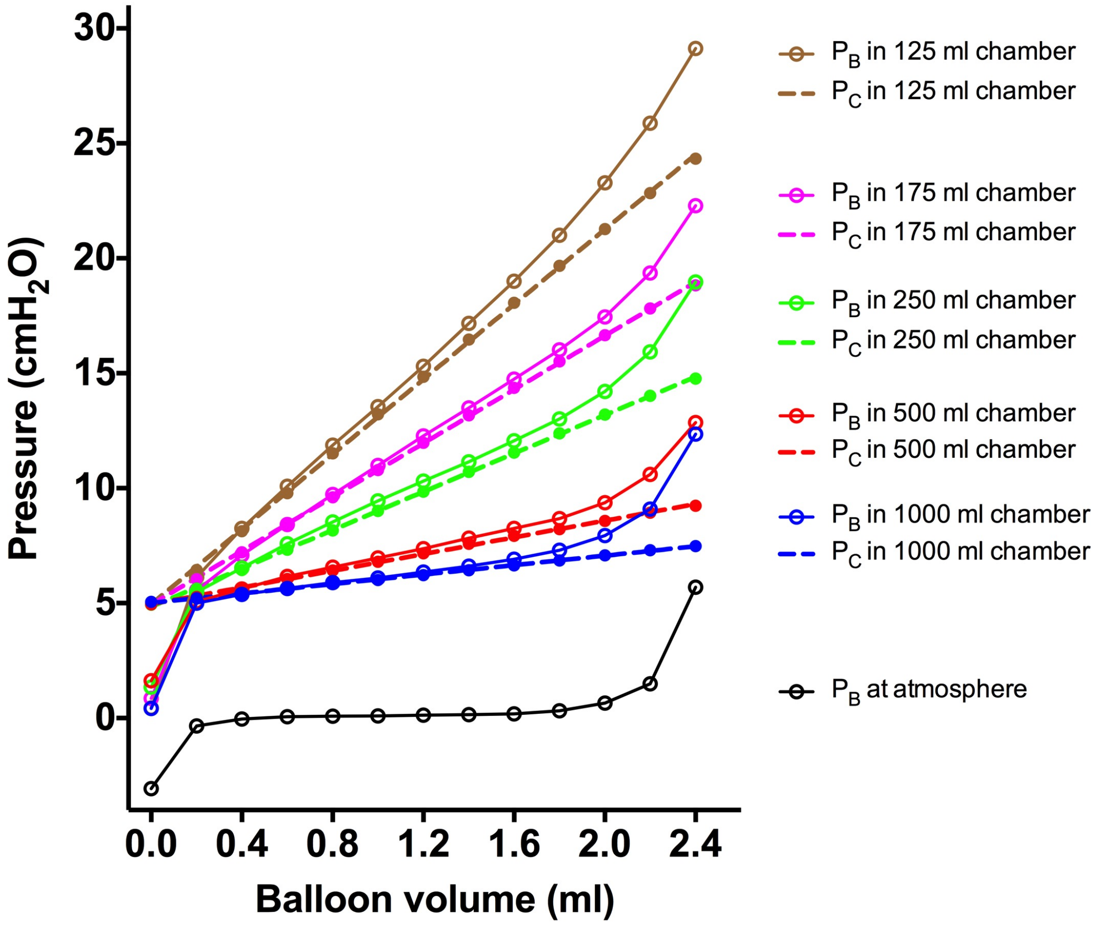

Figure S1. Pressure-volume curves at the atmosphere and in chambers with five different inner volumes at baseline chamber pressure of 5 cmH_2O. Balloon pressure (P_B) is shown as circle and solid line, and chamber pressure (P_C) as dot and dash line. Slope of intermediate linear section increased as chamber volume decreased corresponding to increased chamber elastance.
