# Supplementary material for: Use of esophageal balloon pressure-volume curve analysis to determine esophageal wall elastance and calibrate raw esophageal pressure: a bench experiment and clinical study
Source: BMC Anesthesiol. 2018 Feb 14;18:21. doi: 10.1186/s12871-018-0488-6 (PMC5813414; doi:10.1186/s12871-018-0488-6)
Supplement: Supplementary file 4 — Table S1. Measured chamber elastance (cmH2O/ml) in chambers with different inner volumes. (PDF 62 kb) [file 12871_2018_488_MOESM4_ESM.pdf]

Use of esophageal balloon pressure-volume curve analysis to determine esophageal wall elastance and calibrate raw esophageal pressure: a bench experiment and clinical study

Xiu-Mei Sun, Guang-Qiang Chen, Hua-Wei Huang, Xuan He, Yan-Lin Yang,

Zhong-Hua Shi, Ming Xu, Jian-Xin Zhou

Additional file 4: Table S1

Table S1 Measured chamber elastance (cmH_2O/ml) in chambers with different inner volumes[a]

Baseline chamber pressure	Chamber inner volume				
	1000 ml	500 ml	250 ml	175 ml	125 ml
5 cmH_2O	1.0	1.8	4.2	5.8	8.2
10 cmH_2O	1.0	2.0	4.2	5.9	8.1
15 cmH_2O	1.1	2.0	4.2	5.6	8.0
20 cmH_2O	1.1	1.8	3.9	5.4	7.6
25 cmH_2O	0.9	1.9	3.9	5.2	7.5
30 cmH_2O	0.9	1.7	3.9	5.2	7.5
Median (IQR)	1.0 (0.9–1.1)	1.9 (1.8–2.0)	4.1 (3.9–4.2)	5.5 (5.2–5.8)	7.8 (7.5–8.1)

IQR: interquartile range.

[a]Chamber elastance was measured as the slope of least square fitting between the chamber pressure and balloon volume. Coefficient of determination in least square fitting ranged from 0.987 to > 0.999.
