# Supplementary material for: Use of esophageal balloon pressure-volume curve analysis to determine esophageal wall elastance and calibrate raw esophageal pressure: a bench experiment and clinical study
Source: BMC Anesthesiol. 2018 Feb 14;18:21. doi: 10.1186/s12871-018-0488-6 (PMC5813414; doi:10.1186/s12871-018-0488-6)
Supplement: Supplementary file 5 — Detailed results in the clinical study (PDF 172 kb) [file 12871_2018_488_MOESM5_ESM.pdf]

Use of esophageal balloon pressure-volume curve analysis to determine esophageal wall elastance and calibrate raw esophageal pressure: a bench experiment and clinical study

Xiu-Mei Sun, Guang-Qiang Chen, Hua-Wei Huang, Xuan He, Yan-Lin Yang, Zhong-Hua Shi, Ming Xu, Jian-Xin Zhou

Additional file 5: Detailed results in the clinical study

Balloon volume tests were performed in 40 passive patients under volume-controlled ventilation. The balloon was intermittently inflated in 0.2-ml increment up to 2.4 ml. In some patients, the test was stopped when the balloon was inflated by 1.8 to 2.2 ml because of marked elevation of esophageal balloon pressure. At each tested balloon volume, after 3-min equilibration, airway was occluded at end-expiration and end-inspiration each for 3 s, and esophageal balloon pressure (Pes at EEO and Pes at EIO) was measured. Positive pressure occlusion test was performed at end-expiratory occlusion, and the ratio of changes in esophageal pressure to airway pressure (ΔPes/ΔPaw) during the compression of the chest wall was calculated.

Patient No	Parameters	Balloon volume (ml)												
		0.0	0.2	0.4	0.6	0.8	1.0	1.2	1.4	1.6	1.8	2.0	2.2	2.4
**1**	**Pes at EEO (cmH_2O)**	1.209	7.205	8.282	8.994	9.515	10.477	11.053	11.841	13.203	15.635	25.547	40.700	
	**Pes at EIO (cmH_2O)**	1.541	9.531	10.816	11.771	13.129	14.919	15.945	17.156	18.307	19.781	26.943	41.036	
	**ΔPes/ΔPaw ratio**	0.029	0.506	0.600	0.676	0.813	0.869	0.952	0.929	0.886	0.788	0.341	0.212	
**2**	**Pes at EEO (cmH_2O)**	-0.417	11.713	12.598	12.945	13.554	14.658	15.605	17.619	19.816	22.904	27.321		
	**Pes at EIO (cmH_2O)**	-0.331	13.381	14.456	14.909	15.211	15.951	17.175	18.862	20.779	23.910	27.691		
	**ΔPes/ΔPaw ratio**	0.050	1.124	1.119	1.164	1.092	0.988	1.088	1.094	1.156	1.074	1.034		
**3**	**Pes at EEO (cmH_2O)**	-0.040	5.873	6.391	6.525	7.295	7.880	8.733	9.622	10.900	14.678	32.651	42.212	
	**Pes at EIO (cmH_2O)**	0.405	7.338	8.056	9.222	9.729	10.782	11.969	13.015	13.970	16.551	26.816	41.798	
	**ΔPes/ΔPaw ratio**	0.079	0.539	0.553	0.592	0.784	0.849	0.867	0.877	0.846	0.692	0.283	0.139	
**4**	**Pes at EEO (cmH_2O)**	1.670	9.820	10.631	11.108	11.502	11.933	11.937	12.454	12.467	15.916	27.321		
	**Pes at EIO (cmH_2O)**	1.851	11.425	12.904	14.539	15.462	16.107	16.951	18.202	18.282	20.676	27.691		
	**ΔPes/ΔPaw ratio**	0.047	0.523	0.840	0.817	0.788	0.865	0.886	0.803	0.854	1.131	0.734		
**5**	**Pes at EEO (cmH_2O)**	-0.079	1.929	3.044	4.255	4.219	4.750	5.536	6.894	8.154	11.388	14.954	21.654	34.734
	**Pes at EIO (cmH_2O)**	2.091	7.669	8.331	9.036	9.234	9.877	10.126	10.882	12.403	14.566	16.401	23.680	35.059
	**ΔPes/ΔPaw ratio**	0.082	0.879	0.847	1.069	1.023	0.964	0.918	0.867	0.785	0.796	0.318	0.212	0.043
**6**	**Pes at EEO (cmH_2O)**	0.063	5.125	6.213	6.903	7.704	7.692	9.322	10.777	11.635	13.765	14.482	20.762	32.697
	**Pes at EIO (cmH_2O)**	0.767	9.513	10.372	11.179	11.342	11.272	12.403	13.828	14.227	16.540	17.635	22.598	33.334
	**ΔPes/ΔPaw ratio**	0.156	1.156	1.169	1.097	1.220	1.002	1.194	1.159	1.227	1.148	1.133	1.203	0.224
**7**	**Pes at EEO (cmH_2O)**	0.500	7.730	8.445	8.814	8.928	9.499	10.005	11.059	11.610	12.934	17.784	24.480	37.459
	**Pes at EIO (cmH_2O)**	1.005	10.253	11.261	11.717	11.782	12.444	12.764	13.917	13.921	15.542	20.050	26.387	38.251
	**ΔPes/ΔPaw ratio**	0.136	0.947	1.023	0.990	1.175	1.180	1.181	1.189	1.162	1.166	0.966	0.804	0.325
**8**	**Pes at EEO (cmH_2O)**	0.284	3.310	3.704	3.855	4.164	4.504	4.752	5.431	5.935	6.974	9.312	17.775	32.200
	**Pes at EIO (cmH_2O)**	1.527	5.533	6.103	6.497	7.051	7.647	8.091	8.730	9.344	10.033	11.786	18.756	32.482
	**ΔPes/ΔPaw ratio**	0.317	0.825	0.935	0.991	1.032	0.978	0.964	1.044	1.038	1.095	0.966	0.711	0.289
**9**	**Pes at EEO (cmH_2O)**	0.030	1.587	2.253	2.691	3.156	3.623	4.236	5.354	6.462	8.080	12.363	21.219	34.217
	**Pes at EIO (cmH_2O)**	2.177	4.278	5.294	5.999	6.511	6.795	7.196	7.738	8.552	9.954	13.256	20.946	34.019
	**ΔPes/ΔPaw ratio**	0.583	0.859	0.968	0.975	1.007	1.042	1.025	0.987	0.996	0.840	0.916	0.353	0.191
**10**	**Pes at EEO (cmH_2O)**	0.618	4.324	5.159	5.572	5.995	6.643	7.024	7.590	7.991	8.800	10.782	18.345	
	**Pes at EIO (cmH_2O)**	1.709	6.449	7.456	8.147	9.079	10.263	11.130	12.013	12.433	13.301	14.999	20.307	
	**ΔPes/ΔPaw ratio**	0.244	0.959	0.983	0.966	0.985	1.046	1.019	0.977	0.970	1.002	0.984	0.663	0.280
**11**	**Pes at EEO (cmH_2O)**	0.696	11.010	12.223	13.000	13.994	15.554	16.738	18.087	18.660	21.444	22.543	24.798	27.793
	**Pes at EIO (cmH_2O)**	0.854	13.376	15.155	16.051	16.719	18.187	19.105	19.865	21.024	22.708	24.232	26.426	30.266
	**ΔPes/ΔPaw ratio**	0.046	0.796	0.904	1.055	1.053	1.000	1.036	0.979	1.006	1.003	0.977	0.958	0.690
**12**	**Pes at EEO (cmH_2O)**	-0.022	2.450	2.949	2.974	3.504	4.166	4.888	6.124	9.658	15.965			
	**Pes at EIO (cmH_2O)**	1.105	4.642	5.403	5.499	6.024	6.543	7.186	8.042	10.945	15.728			
	**ΔPes/ΔPaw ratio**	0.379	0.792	0.988	0.847	0.818	0.833	0.900	1.000	0.872	0.759			
**13**	**Pes at EEO (cmH_2O)**	0.457	3.874	4.267	4.805	4.908	5.300	5.834	6.908	8.728	11.862	17.720		
	**Pes at EIO (cmH_2O)**	1.260	6.062	6.953	7.203	7.481	8.040	8.442	9.338	10.636	12.890	17.754		
	**ΔPes/ΔPaw ratio**	0.257	1.049	0.837	0.836	0.861	0.952	0.812	0.832	0.993	0.885	0.696		
**14**	**Pes at EEO (cmH_2O)**	0.814	5.475	6.106	6.870	7.163	7.476	7.530	8.125	9.211	11.560	16.188		
	**Pes at EIO (cmH_2O)**	1.175	7.092	8.176	9.169	9.447	9.634	9.816	10.797	11.922	13.955	17.628		
	**ΔPes/ΔPaw ratio**	0.110	0.505	0.626	0.846	0.832	0.855	0.879	0.914	0.969	1.045	1.137		

Patient No	Parameters	Balloon volume (ml)												
		0.0	0.2	0.4	0.6	0.8	1.0	1.2	1.4	1.6	1.8	2.0	2.2	2.4
**15**	**Pes at EEO (cmH_2O)**	0.640	6.533	7.507	8.156	9.265	9.814	10.992	11.893	13.640	16.535			
	**Pes at EIO (cmH_2O)**	0.942	7.779	9.281	10.354	11.097	11.468	12.372	13.125	14.482	16.841			
	**ΔPes/ΔPaw ratio**	0.166	0.909	0.909	0.919	0.976	0.952	0.938	0.993	0.894	0.684			
**16**	**Pes at EEO (cmH_2O)**	1.703	7.797	8.222	8.382	9.114	9.611	10.126	11.546	13.062	15.202	20.253		
	**Pes at EIO (cmH_2O)**	1.965	9.152	10.076	10.283	11.168	12.080	12.542	13.573	14.800	16.590	20.379		
	**ΔPes/ΔPaw ratio**	0.072	0.828	0.976	0.897	0.867	0.898	0.948	0.916	0.788	0.674	0.516		
**17**	**Pes at EEO (cmH_2O)**	1.130	6.558	7.320	7.893	8.843	9.087	10.140	11.378	12.890	20.439			
	**Pes at EIO (cmH_2O)**	1.367	7.620	8.346	9.643	10.600	10.685	11.833	12.711	14.200	20.612			
	**ΔPes/ΔPaw ratio**	0.078	0.903	0.869	0.903	0.939	0.943	0.940	0.944	0.897	0.660			
**18**	**Pes at EEO (cmH_2O)**	1.634	5.979	7.216	8.241	9.175	9.028	9.810	10.791	12.010	15.932	20.670		
	**Pes at EIO (cmH_2O)**	1.908	6.945	8.667	9.736	11.222	11.132	11.487	12.605	13.910	16.616	20.613		
	**ΔPes/ΔPaw ratio**	0.146	0.804	0.943	0.983	0.995	1.020	1.048	0.937	0.928	0.803	0.564		
**19**	**Pes at EEO (cmH_2O)**	2.877	7.774	8.593	9.002	9.949	10.174	10.797	11.585	12.878	15.620	20.175		
	**Pes at EIO (cmH_2O)**	3.253	10.128	10.690	11.749	13.276	13.221	13.965	14.540	15.613	17.819	21.915		
	**ΔPes/ΔPaw ratio**	0.280	0.768	0.903	1.031	1.023	0.977	1.019	0.967	0.915	0.824	0.614		
**20**	**Pes at EEO (cmH_2O)**	-0.389	1.111	1.802	2.594	3.861	4.973	4.980	5.683	6.844	7.851	8.215	12.410	
	**Pes at EIO (cmH_2O)**	1.273	3.040	4.009	4.742	5.800	6.839	6.948	7.770	8.645	9.339	10.113	13.885	
	**ΔPes/ΔPaw ratio**	0.624	0.743	0.828	0.838	0.846	0.973	0.953	0.949	0.962	1.168	1.022	1.041	
**21**	**Pes at EEO (cmH_2O)**	0.360	2.980	4.212	5.134	5.400	6.220	7.296	7.865	9.517	12.220	20.246		
	**Pes at EIO (cmH_2O)**	1.150	4.782	5.937	6.987	7.263	8.194	9.183	10.059	11.857	14.441	21.952		
	**ΔPes/ΔPaw ratio**	0.300	0.779	0.881	0.959	0.870	1.070	1.135	1.163	1.145	1.130	0.865	0.456	
**22**	**Pes at EEO (cmH_2O)**	0.184	2.669	3.424	3.439	3.680	4.015	4.877	5.961	6.365	7.265	9.124	14.790	
	**Pes at EIO (cmH_2O)**	1.425	4.351	5.272	5.434	5.874	6.239	6.824	7.911	8.284	9.450	11.079	15.779	
	**ΔPes/ΔPaw ratio**	0.633	0.802	0.890	0.877	1.000	1.078	1.138	1.113	1.163	1.228	0.680		
**23**	**Pes at EEO (cmH_2O)**	0.189	4.308	5.121	5.703	5.906	6.074	6.386	6.650	7.435	8.368	10.481	14.843	
	**Pes at EIO (cmH_2O)**	0.577	5.900	6.856	7.417	7.606	7.962	8.263	8.645	9.297	10.593	12.886	16.987	
	**ΔPes/ΔPaw ratio**	0.216	0.748	0.862	0.882	0.997	1.032	1.031	1.080	1.172	1.318	0.582		
**24**	**Pes at EEO (cmH_2O)**	0.343	2.062	2.884	3.451	4.208	5.089	5.808	6.135	6.383	7.449	9.330	13.686	
	**Pes at EIO (cmH_2O)**	1.534	4.160	5.057	5.675	6.232	7.020	7.805	7.977	8.481	9.669	11.134	15.041	
	**ΔPes/ΔPaw ratio**	0.524	0.824	0.758	0.920	0.913	1.056	1.010	1.009	1.143	1.098	1.218	1.097	
**25**	**Pes at EEO (cmH_2O)**	0.688	4.767	5.749	5.822	5.870	6.368	6.741	7.226	7.791	8.672	9.936	12.947	
	**Pes at EIO (cmH_2O)**	1.077	6.374	7.481	7.561	8.100	8.549	8.865	9.353	10.266	11.228	12.966	16.306	
	**ΔPes/ΔPaw ratio**	0.222	0.818	0.848	0.943	0.972	0.983	1.023	1.016	1.142	1.075	1.003	0.957	
**26**	**Pes at EEO (cmH_2O)**	0.490	9.800	11.140	12.120	12.670	13.340	14.490	15.560	16.150	16.940	18.220	20.049	
	**Pes at EIO (cmH_2O)**	1.070	13.380	14.510	15.220	15.940	16.280	17.020	17.780	18.300	18.840	20.390	22.040	
	**ΔPes/ΔPaw ratio**	0.121	0.518	0.967	0.746	0.833	1.007	1.215	1.245	1.299	1.076	0.915	0.959	
**27**	**Pes at EEO (cmH_2O)**	0.210	9.640	10.990	12.200	12.870	13.780	14.690	15.340	16.500	17.480	18.940	21.440	25.984
	**Pes at EIO (cmH_2O)**	0.740	13.170	14.480	15.420	16.220	16.700	17.110	17.700	18.430	19.490	20.620	22.580	26.370
	**ΔPes/ΔPaw ratio**	0.150	0.690	0.827	1.178	0.997	1.089	1.153	1.494	1.474	1.569	1.163	0.869	0.823
**28**	**Pes at EEO (cmH_2O)**	0.700	9.850	11.450	12.080	12.740	13.500	14.310	15.680	16.280	17.060	18.320	20.660	
	**Pes at EIO (cmH_2O)**	1.290	13.220	14.900	15.290	16.110	16.620	17.050	17.570	18.400	19.120	20.240	22.150	
	**ΔPes/ΔPaw ratio**	0.778	0.789	0.837	0.982	0.905	1.030	1.315	1.368	1.405	1.270	1.115	0.733	

Patient No	Parameters	Balloon volume (ml)												
		0.0	0.2	0.4	0.6	0.8	1.0	1.2	1.4	1.6	1.8	2.0	2.2	2.4
**29**	**Pes at EEO (cmH_2O)**	0.130	9.760	11.520	12.150	13.080	14.080	14.930	15.770	16.360	17.380	18.590	20.720	25.910
	**Pes at EIO (cmH_2O)**	0.570	13.120	14.900	15.820	16.150	17.030	17.420	18.340	18.800	19.350	20.550	22.360	26.270
	**ΔPes/ΔPaw ratio**	0.070	0.706	0.783	0.728	0.967	0.934	1.352	1.333	1.194	1.100	0.658	1.187	0.835
**30**	**Pes at EEO (cmH_2O)**	1.090	10.520	11.310	12.560	13.250	14.050	14.750	15.500	16.240	17.080	17.910	19.000	23.120
	**Pes at EIO (cmH_2O)**	1.740	13.430	14.660	15.770	16.690	16.940	17.120	17.960	18.960	19.610	20.050	21.200	24.490
	**ΔPes/ΔPaw ratio**	0.094	0.746	0.806	0.871	0.905	0.837	0.825	1.007	1.264	1.171	1.230	0.963	0.757
**31**	**Pes at EEO (cmH_2O)**	0.520	8.360	10.390	11.650	12.050	12.420	13.120	13.730	14.000	14.960	16.800	20.100	25.930
	**Pes at EIO (cmH_2O)**	1.100	11.080	13.860	15.000	15.560	15.840	16.400	17.270	17.720	18.240	20.120	23.320	27.670
	**ΔPes/ΔPaw ratio**	0.092	0.540	0.785	0.820	0.913	0.809	0.864	0.955	0.833	0.894	0.782	0.747	0.593
**32**	**Pes at EEO (cmH_2O)**	0.150	12.930	14.490	14.960	15.520	15.980	16.390	16.830	17.730	18.680	20.060	22.570	26.720
	**Pes at EIO (cmH_2O)**	0.260	14.310	16.750	17.380	17.730	18.290	17.710	19.180	19.630	20.650	22.340	24.080	27.620
	**ΔPes/ΔPaw ratio**	0.028	0.354	0.824	0.853	1.087	0.952	0.855	0.847	0.928	0.894	0.879	0.856	0.647
**33**	**Pes at EEO (cmH_2O)**	0.460	13.430	14.880	15.090	15.520	16.270	16.540	17.230	18.150	19.230	20.090	23.140	27.330
	**Pes at EIO (cmH_2O)**	0.590	14.860	16.980	17.290	18.130	18.600	19.130	19.520	20.030	21.110	22.580	24.780	28.270
	**ΔPes/ΔPaw ratio**	0.035	0.290	0.942	0.963	0.988	0.975	0.939	0.932	0.959	0.870	0.879	0.725	0.743
**34**	**Pes at EEO (cmH_2O)**	0.790	13.580	15.050	15.570	16.110	16.600	17.110	17.760	18.360	19.460	20.860	23.070	27.390
	**Pes at EIO (cmH_2O)**	0.920	14.820	17.380	17.750	18.170	19.030	19.440	20.110	20.600	21.770	22.560	24.950	28.490
	**ΔPes/ΔPaw ratio**	0.039	0.303	0.869	0.943	0.984	1.004	0.934	0.981	0.868	0.885	0.863	0.845	0.680
**35**	**Pes at EEO (cmH_2O)**	0.600	13.260	14.860	15.330	15.740	16.050	16.710	17.630	18.440	19.480	20.830	23.350	27.600
	**Pes at EIO (cmH_2O)**	0.730	14.490	17.350	17.290	18.100	18.590	19.140	19.690	20.590	21.340	22.620	24.780	28.570
	**ΔPes/ΔPaw ratio**	0.081	0.305	0.813	0.874	0.902	0.929	0.940	1.016	0.932	0.942	0.803	0.829	0.717
**36**	**Pes at EEO (cmH_2O)**	0.260	11.510	13.440	14.690	15.510	16.170	16.830	17.860	19.130	20.710	22.830	25.370	
	**Pes at EIO (cmH_2O)**	0.430	14.620	17.200	17.940	18.250	18.570	19.350	19.970	21.060	22.130	23.720	26.160	
	**ΔPes/ΔPaw ratio**	0.056	0.695	1.252	1.235	1.171	1.156	1.196	1.005	1.069	1.125	0.944	0.856	
**37**	**Pes at EEO (cmH_2O)**	0.440	8.410	9.260	9.990	10.500	10.990	12.410	13.050	14.080	15.260	17.370	21.650	28.170
	**Pes at EIO (cmH_2O)**	0.950	10.790	12.100	13.050	13.660	14.530	15.450	16.260	17.050	18.360	20.490	23.870	29.280
	**ΔPes/ΔPaw ratio**	0.113	0.643	0.774	0.821	0.809	0.882	0.887	0.908	0.837	0.857	0.778	0.662	0.442
**38**	**Pes at EEO (cmH_2O)**	0.610	7.990	9.850	10.930	11.560	11.870	12.560	13.360	14.030	14.780	16.860	20.210	26.140
	**Pes at EIO (cmH_2O)**	1.200	10.620	12.800	14.270	14.900	15.220	15.750	16.620	17.090	17.810	19.900	22.690	27.950
	**ΔPes/ΔPaw ratio**	0.108	0.609	0.680	0.716	0.837	0.844	0.819	0.883	0.873	0.832	0.782	0.698	0.525
**39**	**Pes at EEO (cmH_2O)**	0.450	8.970	9.860	10.490	10.940	11.560	12.890	13.450	14.280	15.430	17.390	19.840	24.610
	**Pes at EIO (cmH_2O)**	0.830	11.410	13.240	13.760	14.430	15.060	15.980	16.760	17.640	18.740	20.450	22.610	26.640
	**ΔPes/ΔPaw ratio**	0.065	0.566	0.761	0.835	0.860	0.831	0.845	0.800	0.950	0.884	0.874	0.759	0.560
**40**	**Pes at EEO (cmH_2O)**	0.530	12.370	13.700	14.490	15.340	15.740	16.130	16.640	17.530	18.510	19.980	22.360	26.550
	**Pes at EIO (cmH_2O)**	0.680	14.090	16.330	16.940	17.550	18.190	18.650	19.210	19.700	20.500	21.590	23.750	27.280
	**ΔPes/ΔPaw ratio**	0.042	0.391	0.841	0.893	0.968	0.915	0.875	0.848	0.911	0.906	0.931	0.784	0.684
