# Supplementary material for: Use of esophageal balloon pressure-volume curve analysis to determine esophageal wall elastance and calibrate raw esophageal pressure: a bench experiment and clinical study
Source: BMC Anesthesiol. 2018 Feb 14;18:21. doi: 10.1186/s12871-018-0488-6 (PMC5813414; doi:10.1186/s12871-018-0488-6)
Supplement: Supplementary file 6 — Table S2. Individual data of balloon volume and esophageal wall elastance. (PDF 71 kb) [file 12871_2018_488_MOESM6_ESM.pdf]

Use of esophageal balloon pressure-volume curve analysis to determine esophageal wall elastance and calibrate raw esophageal pressure: a bench experiment and clinical study

Xiu-Mei Sun, Guang-Qiang Chen, Hua-Wei Huang, Xuan He, Yan-Lin Yang,

Zhong-Hua Shi, Ming Xu, Jian-Xin Zhou

Additional file 6: Table S2

Table S2 Individual data of balloon volume and esophageal wall elastance

No of Patient	V_{MIN} (ml)	V_{MAX} (ml)	V_{BEST} (ml)	Ees by standard method (cmH_2O/ml)	Ees by simple method (cmH_2O/ml)
1	0.2	1.4	1.4	3.7	3.6
2	0.2	1.4	0.6	3.7	3.3
3	0.2	1.6	1.4	3.5	3.9
4	0.2	1.6	1.4	1.8	1.7
5	0.6	1.2	1.0	3.3	3.3
6	0.4	1.6	0.6	4.5	4.8
7	0.2	1.6	1.0	2.6	2.8
8	0.2	1.4	1.2	1.6	2.0
9	0.4	1.4	0.8	2.4	1.9
10	0.4	1.4	1.4	2.5	2.5
11	0.2	1.6	0.6	5.8	5.6
12	0.2	1.4	0.6	2.9	2.4
13	0.2	1.4	1.0	1.9	2.6
14	0.2	1.4	1.4	2.0	1.6
15	0.2	1.4	0.6	4.4	4.7
16	0.4	1.4	1.2	2.4	2.4
17	0.2	1.6	0.8	4.3	4.4
18	0.2	1.6	1.0	3.8	3.2
19	0.2	1.4	1.2	3.0	3.2
20	0.2	2.0	0.6	4.0	3.9
21	0.4	1.4	1.4	3.7	3.4
22	0.2	1.8	1.0	2.8	3.2
23	0.6	1.4	1.4	1.2	1.2
24	0.2	1.6	0.6	3.2	3.4
25	0.4	1.4	0.8	1.8	1.8
26	0.6	2.0	0.8	4.4	4.3
27	0.6	2.0	0.8	4.7	4.2
28	0.6	2.0	0.8	4.3	4.5
29	0.4	2.0	0.6	4.4	4.5
30	0.2	2.2	0.8	4.1	3.7
31	0.6	1.4	1.4	2.5	2.6
32	0.4	1.4	1.2	2.4	2.3
33	0.4	2.0	0.8	3.3	2.7
34	0.4	1.6	1.0	2.7	2.7
35	0.4	1.6	1.0	2.9	2.9
36	0.6	1.4	0.6	3.8	4.0
37	0.2	1.8	1.0	4.2	3.8
38	0.6	1.8	1.0	3.2	3.0
39	0.2	1.8	1.0	3.9	3.7
40	0.6	1.8	1.4	3.1	2.7
Median (IQR)	0.3 (0.2–0.4)	1.6 (1.4–1.8)	1.0 (0.8–1.2)	3.3 (2.5–4.1)	3.2 (2.6–3.9)

IQR: interquartile range, Ees: esophageal wall elastance, V_{MIN} and V_{MAX}: the minimal and maximal balloon volume representing the optimal filling volume with the least influence of balloon recoil pressure, V_{BEST}: the best balloon filling volume.
