# Supplementary material for: Use of esophageal balloon pressure-volume curve analysis to determine esophageal wall elastance and calibrate raw esophageal pressure: a bench experiment and clinical study
Source: BMC Anesthesiol. 2018 Feb 14;18:21. doi: 10.1186/s12871-018-0488-6 (PMC5813414; doi:10.1186/s12871-018-0488-6)
Supplement: Supplementary file 7 — Figure S2. Examples of in vivo esophageal balloon pressure-volume curve during end-expiratory occlusion in combination with in vitro curve in chamber with comparable elastance and baseline pressure. (PDF 571 kb) [file 12871_2018_488_MOESM7_ESM.pdf]

Use of esophageal balloon pressure-volume curve analysis to determine esophageal wall elastance and calibrate raw esophageal pressure: a bench experiment and clinical study

Xiu-Mei Sun, Guang-Qiang Chen, Hua-Wei Huang, Xuan He, Yan-Lin Yang,

Zhong-Hua Shi, Ming Xu, Jian-Xin Zhou

Additional file 7: Figure S2

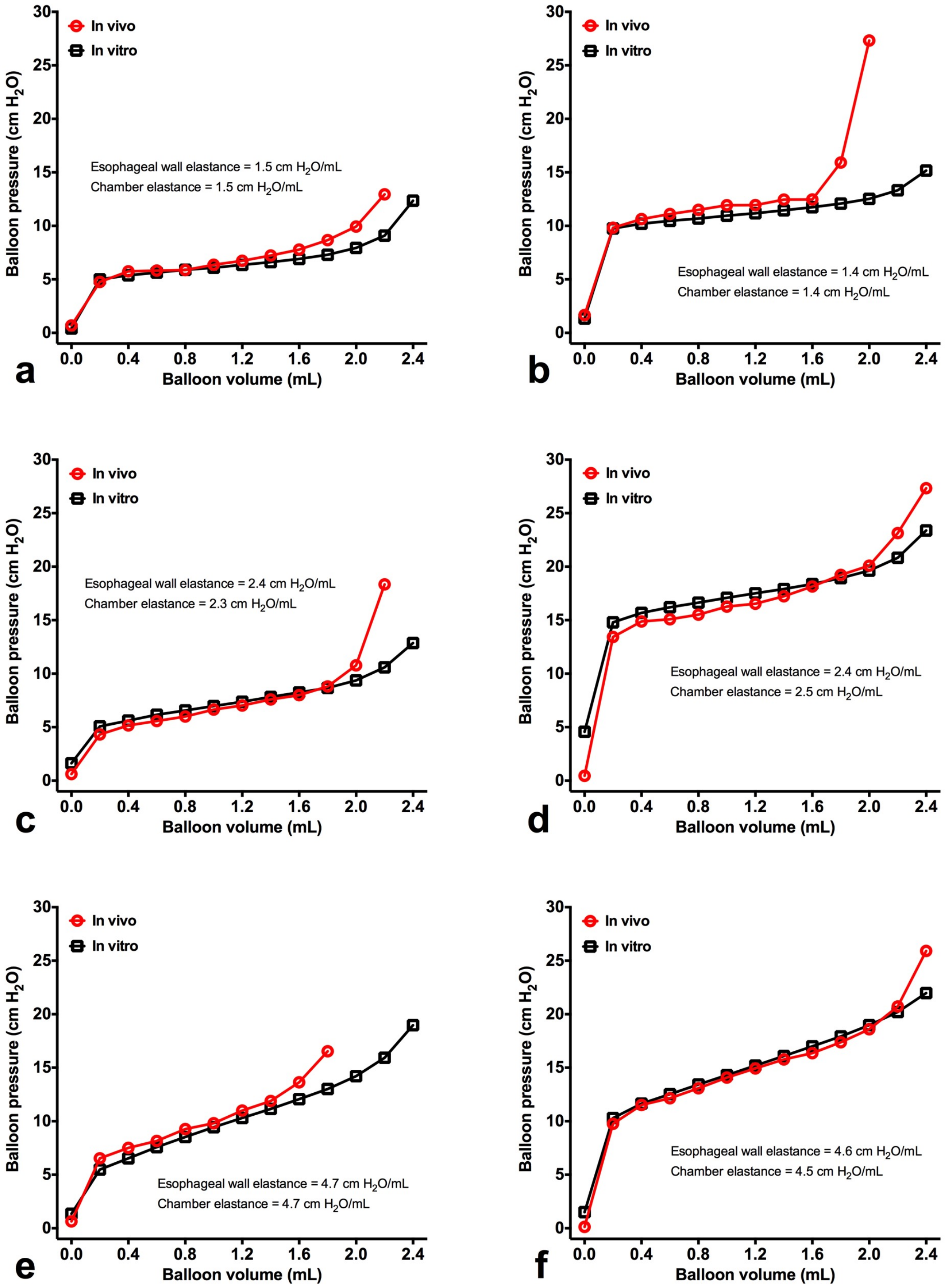

Figure S2. Examples of in vivo esophageal balloon pressure-volume curve (red) during end-expiratory occlusion in combination with in vitro curve (blace) in chamber with comparable elastance and baseline pressure. Estimated esophageal elastance and chamber elastance are shown. Bench balloon pressure-volume curves are presented in chambers with inner volume of 1000ml at baseline pressure of 5 cmH_2O (panel **a**) and 10 cmH_2O (panel **b**), inner volume of 500 ml at baseline pressure of 5 cmH_2O (panel **c**) and 15 cmH_2O (panel **d**), and inner volume of 250 ml at baseline pressure of 10 cmH_2O (panel **e**) and 15 cmH_2O (panel **f**).
