# Supplementary material for: Use of esophageal balloon pressure-volume curve analysis to determine esophageal wall elastance and calibrate raw esophageal pressure: a bench experiment and clinical study
Source: BMC Anesthesiol. 2018 Feb 14;18:21. doi: 10.1186/s12871-018-0488-6 (PMC5813414; doi:10.1186/s12871-018-0488-6)
Supplement: Supplementary file 8 — Figure S3. Bland-Altman’s limits of agreements analysis for the estimated esophageal elastance by the simple and standard linear regression method. (PDF 287 kb) [file 12871_2018_488_MOESM8_ESM.pdf]

Use of esophageal balloon pressure-volume curve analysis to determine esophageal wall elastance and calibrate raw esophageal pressure: a bench experiment and clinical study

Xiu-Mei Sun, Guang-Qiang Chen, Hua-Wei Huang, Xuan He, Yan-Lin Yang,

Zhong-Hua Shi, Ming Xu, Jian-Xin Zhou

Additional file 8: Figure S3

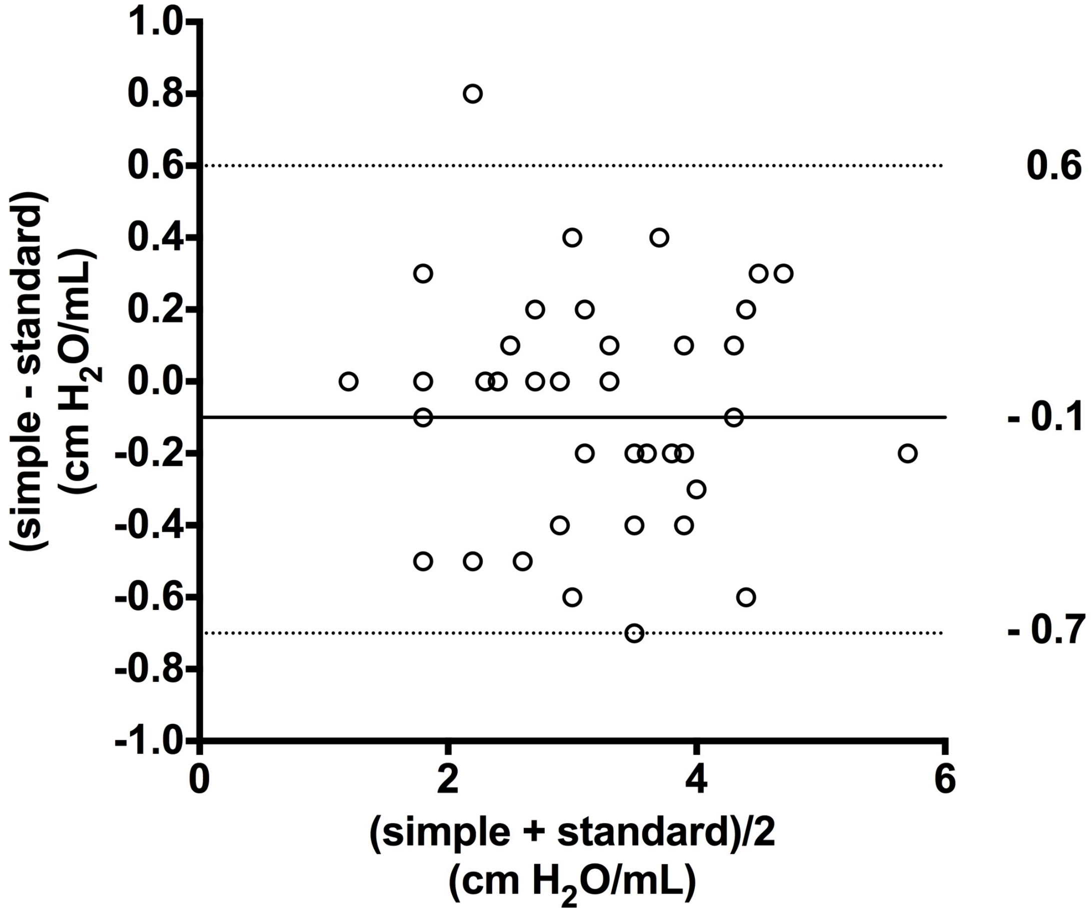

Figure S3. Bland-Altman's limits of agreements analysis for the estimated esophageal elastance by the simple and standard linear regression method. In the standard method, the slope of intermediate linear section on end-expiratory balloon pressure-volume was used to estimate the esophageal wall elastance. In the simple method, the esophageal wall elastance was estimated by the difference of end-expiratory balloon pressure at 0.6 ml and 1.4 ml divided by 0.8 ml. Bias (solid line) and upper and lower limits of agreements (dash lines) are shown.
